# Distinct roles of amniotic membrane epithelial (hAEC) and mesenchymal stromal cells (hAMSC) in amniotic membrane-driven wound healing

**DOI:** 10.1038/s41598-025-20685-4

**Published:** 2025-10-21

**Authors:** M. Alcaraz, I. Hernández-Mármol, J. M. Puente-Cuadrado, M. Rodríguez-Valiente, A. R. Silini, O. Parolini, F. J. Nicolás

**Affiliations:** 1 Soluciones de biología molecular y celular en medicina regenerativa, Health Sciences PhD Program, Universidad Católica de Murcia (UCAM), Campus de los Jerónimos no 135, Guadalupe, Murcia, 30107 Spain; 2 Regeneración Oncología Molecular y TGF-β, Trasplantehematopoyético/terapia celular, Instituto Murciano de Investigación Biosanitaria Pascual Parrilla-IMIB, Murcia, 30120 Spain; 3 Instituto Murciano de Investigación Biosanitaria Pascual Parrilla-IMIB, El Palmar, Murcia, 30120, Spain; 4 Centro di Ricerca E. Menni, Fondazione Poliambulanza Istituto Ospedaliero, Brescia, 25124 Italy; 5 Department of Life Science and Public Health, Università Cattolica del Sacro Cuore, Rome, 00168 Italy; 6 Fondazione IRCCS Casa Sollievo della Sofferenza, San Giovanni Rotondo, Foggia, 71013, Italy

**Keywords:** Amniotic membrane, Chronic wounds, Wound healing, TGF-β, hAEC, hAMSC, Conditioned media, Germ cells, Biotechnology, Cell biology, Stem cells

## Abstract

Human skin wound healing is a complex process involving sequential cellular events to restore skin integrity. Chronic wounds fail to heal due to impaired keratinocyte activation and dysregulated cytokine signaling. The human amniotic membrane (hAM), a perinatal derivative, is widely used in hospitals due to its therapeutic effects on wound healing. hAM contains two main cell types: human Amniotic Epithelial Cells (hAEC) and human Amniotic Mesenchymal Stromal Cells (hAMSC). This study isolated and cultured both cell types, using their conditioned media in assays for cell migration, proliferation, and TGF-β cell-cycle arrest rescue. While hAEC-conditioned media only had similar effects to hAM in cell migration, hAMSC-conditioned media demonstrated superior efficacy in this and other assays. These findings suggest that hAMSC are the primary contributors to AM’s beneficial effects in chronic wound healing, highlighting their potential for targeted therapeutic applications.

**Supplementary Information:**

The online version contains supplementary material available at 10.1038/s41598-025-20685-4.

## Introduction

Human skin wound healing is a finely tuned process comprising sequential cellular events that restore skin integrity^1–4^. This reparative process is critical for maintaining skin homeostasis and integrity, as the skin serves as a physical barrier against infections, ultraviolet radiation, and temperature fluctuations^5^. However, complications in wound healing can result in incomplete wound closure, often associated with sustained inflammation, which leads to chronic, non-healing wounds^6,7^. Chronic wounds are a major cause of ulcers—lesions that significantly compromise a patient’s quality of life and are further exacerbated by conditions such as diabetes or aging^8–13^. Indeed, the prevalence of ulcers is higher among the elderly, a demographic increasingly growing due to an increasingly aging society^14^. As a consequence, chronic wounds impose significant financial and logistical burdens on healthcare systems, requiring extensive resources and prolonged treatment^15–17^.

The human amniotic membrane (hAM) the innermost layer of the amniotic sac, is a well-studied perinatal derivative with multiple therapeutic effects, particularly in tissue regeneration^18^. Its application in hospitals is now considered an innovative and effective therapy^18–21^. Previous studies have demonstrated that hAM enhances wound healing both in vitro and in vivo^22,23^. Notably, keratinocyte proliferation and migration—two critical cellular events in wound healing—are significantly enhanced by hAM^3,23,24^. Using immortalized keratinocytes (HaCaT cells), we previously showed that hAM promotes their migration during scratch assays. Moreover, hAM can rescue HaCaT cells from TGF-β-induced cell cycle arrest and restore proliferation^23^. Moreover, hAM increases the motility of epithelial cells by enhancing actin cytoskeleton and focal adhesion turnover^25^. The molecular mechanisms underlying these effects are driven by EGF signaling, involving MAP kinase activation and the regulatory contribution of TGF-β1^26,27^. A key outcome of this signaling is the upregulation of transcription factor c-Jun, a key regulator for cell migration^28–30^. Interestingly, we have shown that hAM produces the reepithelization of complex wounds in vivo, evidenced by successful clinical trials where hAM was used in patients with deep traumatic wounds and diabetic foot ulcer (DFU)^19,22,23,31,32^. Strikingly, we have observed that hAM induces overexpression of c-Jun, which promotes wound re-epithelialization^22,23,33^. hAM is particularly effective in chronic, non-healing wounds, where it stimulates cellular and molecular processes impaired by the chronic pro-inflammatory tissue environment^24,26,27^. To reproduce this chronic wound context in vitro, we recently developed an in vitro model where HaCaT cells were serum-starved and simultaneously treated with TGF-β1 for 48 h. These cells exhibit an altered phenotype with a characteristic genetic profile, including cell cycle arrest, impaired migration, altered morphology, and increased expression of genes related to senescence and inflammation^34^. We have called these Serum Starved TGF-β Chronified (SSTC)-HaCaT cells^34^. Interestingly, hAM treatment of SSTC-HaCaT cells restores their migratory potential, improves cytoskeletal dynamics, and rescues their proliferative capacity while reducing markers of senescence and inflammation^33^. Therefore, these findings compile the therapeutic features of hAM in promoting wound healing through various mechanisms.

Given its unique properties, hAM is a promising treatment for chronic, non-healing wounds. However, it is important to note that hAM collection and processing require specialized facilities, such as tissue banks equipped with cleanrooms, which are often limited to major reference hospitals^35,36^.

hAM cells are of fetal origin^37^ and are organized into two main and unique populations: human amniotic epithelial cells (hAEC), which are in direct contact with the amniotic fluid, and human amniotic membrane mesenchymal stromal cells (hAMSC), which are embedded in the underlying connective tissue^37^. These cells can be isolated either as a heterogeneous population or separately through enzymatic digestion. Typically, protocols involve using trypsin to isolate hAEC and collagenase to isolate hAMSC, though other enzymes, such as dispase or a combination of collagenase/DNase, have also been employed^37^. In our lab, we have previously studied the effects of the whole hAM on various aspects of wound healing, including cell migration, cell proliferation, and TGF-β signalling attenuation^23,25–27,31,33^. The purpose of this research was to investigate the specific contributions of different hAM cell types to the phenomena underlying hAM’s powerful wound-healing properties. To this end, we isolated and purified the two main hAM cell types, hAEC and hAMSC, and tested the effects of their conditioned media—individually and / or in combination—on processes related to wound healing. These effects were then compared to those of the whole hAM to identify which cell types are primarily responsible for hAM’s therapeutic effects.

## Results

### Isolation of hAEC and hAMSC from hAM

**Fig. 1:**
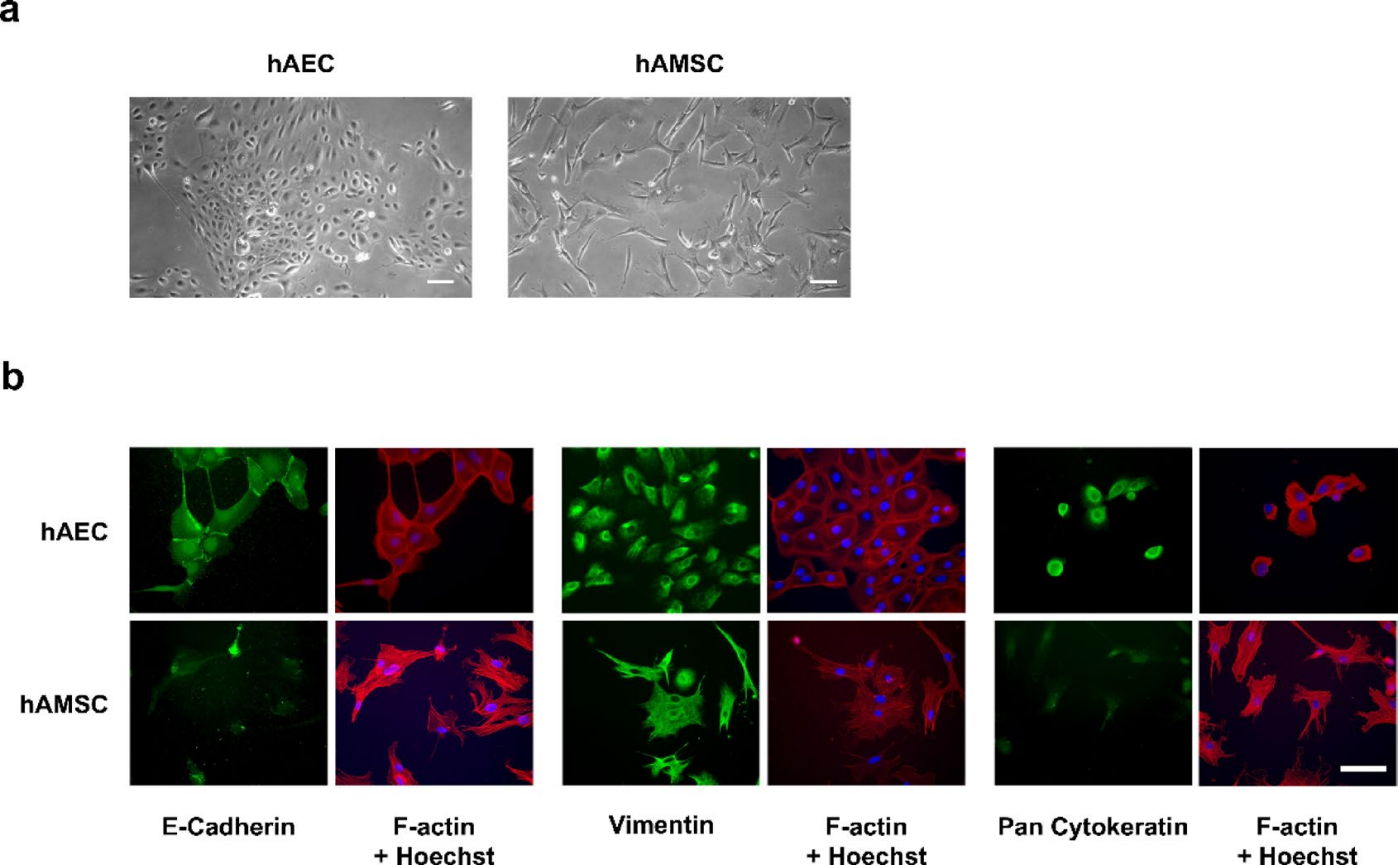
hAEC and hAMSC cells isolated from hAM. hAEC or hAMSC isolated from term amniotic membrane (hAM) were isolated and cultured. (**a**) Phase-contrast images of subconfluent hAECs and hAMSC at P0 are shown, captured at 10x magnification. Scale bar: 50 μm. (**b**) Cultured hAEC or hAMSC at P0 were immunostained for E-cadherin, Vimentin, and Pan-cytokeratins; and co-stained for F-Actin. Nuclei were counterstained with Hoechst 33258. All images were acquired using epifluorescence microscopy. Scale bar: 50 μm. *N*≥3.

hAEC and hAMSC were isolated from the hAM and cultured in the recommended media. Both cell types were analyzed at different passages and show the typical features previously described by many authors^37^. hAEC exhibited a typical cuboidal morphology and a tendency to grow in clusters (Fig. 1a). In contrast, hAMSC displayed a clear mesenchymal morphology from the first passage, growing as independent spindle-shaped cells with a robust proliferation rate (Fig. 1a). After isolation, the cells were characterized for typical mesenchymal and epithelial markers, with the results summarized in Table 1. Both cell types expressed markers consistent with their respective identities, suggesting a high degree of purity in the isolated populations. However, a slight contamination with epithelial cells was detected in hAMSC, that in the next cell passage disappear due to selection with the CHANG medium D©.

For hAEC, E-Cadherin was initially observed at intercellular junctions, indicating epithelial integrity. Interestingly, Vimentin, an antigen not expressed by epithelial cells in the native amniotic membrane^38^, became evident as soon as the cells were cultured. Finally, pan-Cytokeratin was consistently present in hAEC, further confirming their epithelial nature. For hAMSC, E-Cadherin was negative. In contrast, Vimentin expression was evident starting at passage 0. Conversely, pan-Cytokeratin was not detected, further supporting the lack of epithelial contamination in hAMSC cultures after one passage (Fig. 1b).

These findings show that, in our hands, we could isolate the two distinct cell populations from the hAM using the appropriate combination of enzymatic treatments^37^.

**Table 1:** Isolated hAEC and hAMSC were analyzed by flow cytometry for the antigens shown in the table at different passages.

hAEC		
Markers	*P*_0_	*P*_1_
CD73	+	+++
CD90	---	+
CD105	---	---
CD13	---	--
CD66	---	---
E-Cadherin	+++	+
SSEA-4	+++	+++

hAMSC		
Markers	P_0_	P_2_
CD73	--	+++
CD90	-	+++
CD105	---	-
CD13	+++	+++
CD66	--	---
E-Cadherin	+	--
SSEA-4	+++	+

A scale between three negative (---) signs and three positive (+++) signs was used to stablish the intensity of expression.

### CM from both hAEC and hAMSC stimulated migration in human keratinocytes

Our lab has previously shown that the hAM induces keratinocyte migration and counteracts the effects of TGF-β-induced chronification in HaCaT cells^33^. In this study, we sought to determine whether CM derived from hAEC (hAEC-CM) or hAMSC (hAMSC-CM) could replicate the effects observed in HaCaT keratinocytes following hAM treatment. Consistent with our findings, previous studies using transwell membrane inserts demonstrated that the pro-migratory activity of hAM cells is mediated predominantly by soluble factors, rather than direct cell–cell contact^22,23^. We highlight this here to prevent potential misinterpretation.

**Fig. 2:**
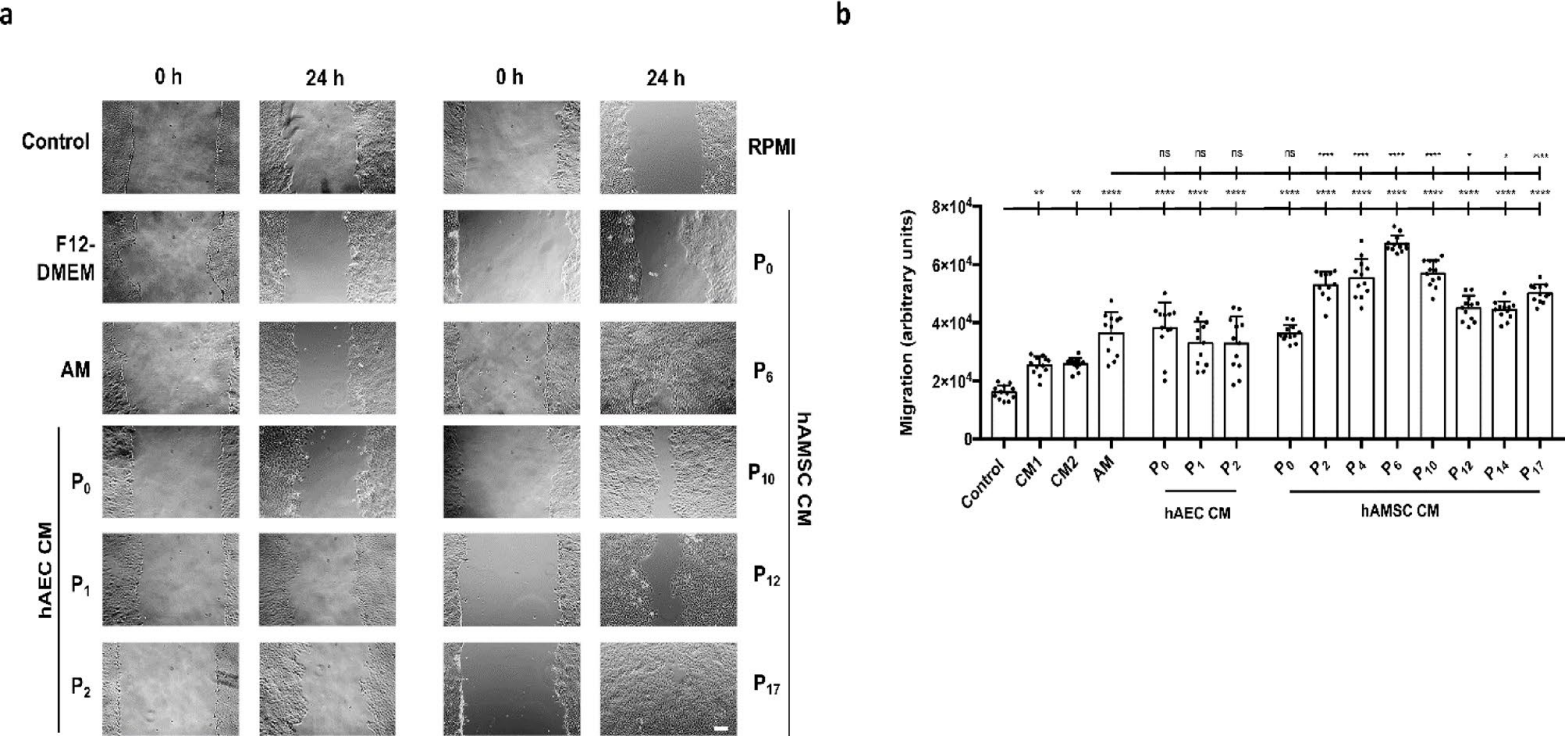
Conditioned media from hAEC and hAMSC enhances cell migration in HaCaT cells. Confluent, serum-starved HaCaT cells (SS-HaCaT) were scratched and treated with either hAM or hAEC-CM or hAMSC-CM for 24 h. F12-DMEM or RPMI (CM1 or CM2 respectively in [**b**]) refers to the media used for hAEC or hAMSC, respectively, for the conditioning. (**a**) Representative images of the wound area before treatment (0 h) and after 24 h of treatment. Scale bar: 200 μm. (**b**) Quantification of cell migration. Each point represents an individual measurement, with the bar indicating the average across all data points. Data were derived from three independent experiments and are presented as mean ± SEM. Asterisks denote statistically significant differences between conditions, determined by one-way ANOVA followed by Tukey’s multiple comparisons test (*p < 0.05, **p < 0.01, ***p < 0.001, ****p < 0.0001; ns: not significant, *p* > 0.05).

To test this, we performed in vitro wound healing scratch assays on serum-starved HaCaT cells (SS-HaCaT). Cells were treated with hAM alone or with hAEC-CM and hAMSC-CM at various passages. The migration analysis clearly showed that cells were stimulated to migrate in the presence of hAM (Fig. 2a). This migration-stimulating capacity was replicated by hAEC-CM (Fig. 2b). Interestingly, when hAEC-CM at different passages were tested, the migration potency remained consistent across passages (Fig. 2b). Because hAEC undergo growth arrest at very early passages, several comparisons with hAMSC were necessarily conducted using non-equivalent passage numbers. While the overall trends remain consistent, we cannot exclude that some of the observed differences may be influenced by passage-dependent changes. Surprisingly, hAMSC-CM exhibited a notable difference compared to hAM. While the CM from freshly isolated hAMSC (P0) showed limited potency, subsequent passages demonstrated significantly stronger migration-stimulating effects, surpassing those of hAEC-CM and even hAM itself. Altogether, these data suggest that both types of CM promoted cell migration under serum-starved conditions. However, hAMSC-CMs were significantly more effective in enhancing migration than hAEC-CMs. Moreover, a particularly robust response was observed in cells treated with hAMSC-CM. Notably, regardless of the passage number of the hAMSC used, except for passage 0, all samples consistently exhibited greater migration-stimulating potency compared to hAM.

### hAMSC CM induced c-Jun expression in HaCaT cells

**Fig. 3:**
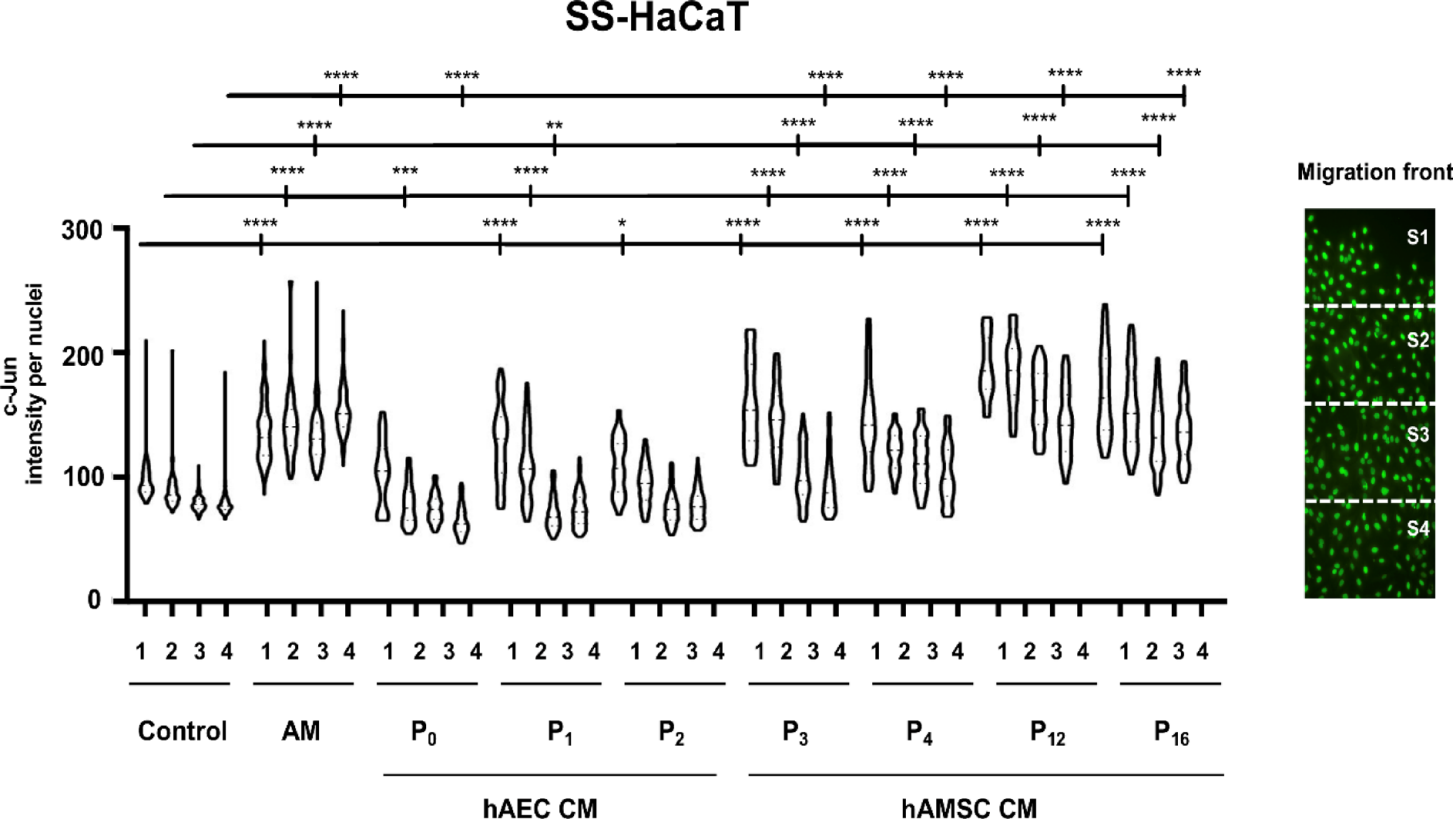
Both hAEC and hAMSC conditioned media enhance the expression of c-Jun at the wound edge of serum-starved HaCaT cells. Confluent serum-starved HaCaT cells (SS-HaCaT) were scratched and treated with hAM or conditioned media (CM) from either hAEC or hAMSC. Cells were allowed to migrate for 24 h and were subsequently immunostained with a specific antibody against c-Jun. The image illustrates how a tile scan is divided into four equal sectors to facilitate the analysis of c-Jun expression. Each point in the plot represents the c-Jun intensity within individual nuclei in the corresponding sector. Asterisks denote statistically significant differences between conditions, as determined by two-way ANOVA followed by Tukey’s multiple comparisons test (ns: *p* > 0.05, *p < 0.05, **p < 0.01, ***p < 0.001, ****p < 0.0001). *N* ≥ 3.

**Fig. 4:**
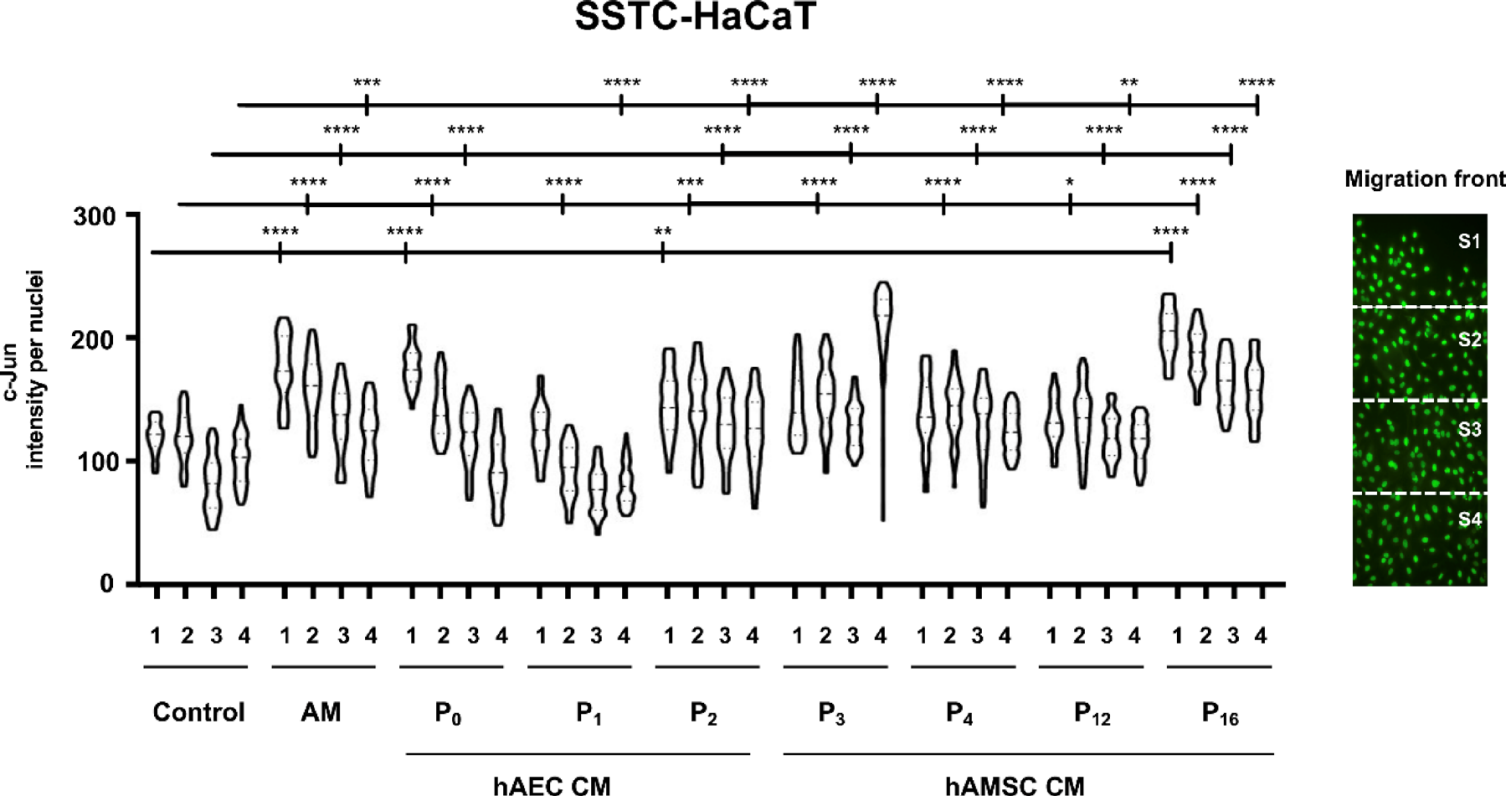
Both hAEC-CM and hAMSC-CM enhance the expression of c-Jun at the wound edge of SSTC-HaCaT cells. Confluent SSTC-HaCaT cells were scratched and treated with hAM or either hAEC-CM or hAMSC-CM. Cells were allowed to migrate for 24 h and were subsequently immunostained with a specific antibody against c-Jun. The image illustrates how a tile scan is divided into four equal sectors to facilitate the analysis of c-Jun expression. Each point in the plot represents the c-Jun intensity within individual nuclei in the corresponding sector. Asterisks indicate statistically significant differences between conditions, as determined by two-way ANOVA followed by Tukey’s multiple comparisons test (ns: *p* > 0.05, *p < 0.05, **p < 0.01, ***p < 0.001, ****p < 0.0001). *N* ≥ 3.

The hAM induces the overexpression of c-Jun at the wound edge of chronic wounds, a phenomenon that is replicated when SSTC-HaCaT cells are treated with hAM^33^. This overexpression of c-Jun correlates strongly with the enhanced cell migration observed^25,33^. To evaluate the ability to induce c-Jun expression at the wound border, confluent cells were scratched and treated with different CM derived from hAEC and hAMSC, as well as with hAM. Under serum-starved (SS) conditions, hAM induced significant c-Jun overexpression at the wound border. In contrast, hAEC-CM did not produce this effect However, when hAMSC-CMs were applied, a strong overexpression of c-Jun was observed at the migrating front, extending deeply into the epithelial layer (Fig. 3). Treatment with hAMSC-CM on SSTC-HaCaT cells similarly induced upregulation of c-Jun, with the expression extending a certain distance from the wound edge. However, hAEC-CM, similarly to hAM, elicited a sustained expression of c-Jun at the wound edge that experience a slight decay to rear nuclei (Fig. 4).

**Fig. 5:**
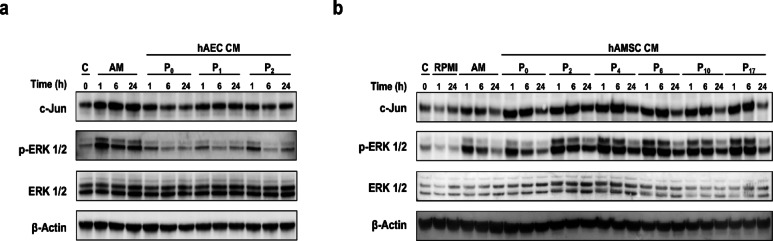
hAEC-CM and hAMSC-CM stimulates phosphorylation of both ERK and c-Jun. (**a**) Total protein extracts from subconfluent, serum-starved HaCaT cells treated with either hAM-CM or hAEC-CM for 1, 6, or 24 h were analyzed by PAGE and western blot. (**b**) Total protein extracts from subconfluent, serum-starved HaCaT cells treated with either hAM or hAMSC-CM for 1, 6, or 24 h were similarly analyzed by PAGE and western blot. In all cases, membranes were probed with antibodies against c-Jun, phospho-ERK1/2, ERK1/2, and phospho-c-Jun. β-actin was used as a loading control. *N* ≥ 3. A representative experiment is shown.

At the molecular level, protein phosphorylation analysis was performed to contrast previous observations. Under confluent cells were stimulated for different times with hAM or CM from cells. The expression and phosphorylation of several proteins was studied by western blot. Although hAEC-CM induced some stimulation of c-Jun expression and ERK phosphorylation (Fig. 5a; Supplemental Fig. 1a), a markedly stronger response was observed when hAMSC-CM were used, even at high passage numbers (e.g., passage 17) (Fig. 5b; Supplemental Fig. 1b). Notably, both c-Jun and p-ERK levels induced by hAMSC-CM exceeded those induced by hAM itself. In contrast, neither c-Jun nor p-ERK were upregulated beyond the levels observed with hAM in cells treated with hAEC-CM.

These findings suggest that the reduced migration of HaCaT cells stimulated with hAEC-CM correlates with the minimal activation of key proteins and signaling pathways essential for cell migration, that in contrast is maximally activated by the presence of hAMSC and is coherent with its maximum potency on cell migration.

### The mixture of hAEC-CM and hAMSC-CM did not exhibit any distinctive features beyond those observed with hAMSC-conditioned media alone

Given that hAM is composed almost exclusively of hAEC and hAMSC^37^, and that hAM demonstrates significant positive effects on migration and associated protein activation, we decided to evaluate the impact of combining CM on cell systems to assess potency. A mixture of hAEC-CM and hAMSC-CM (1:1 ratio) was tested and compared to single-origin media or hAM.

**Fig. 6:**
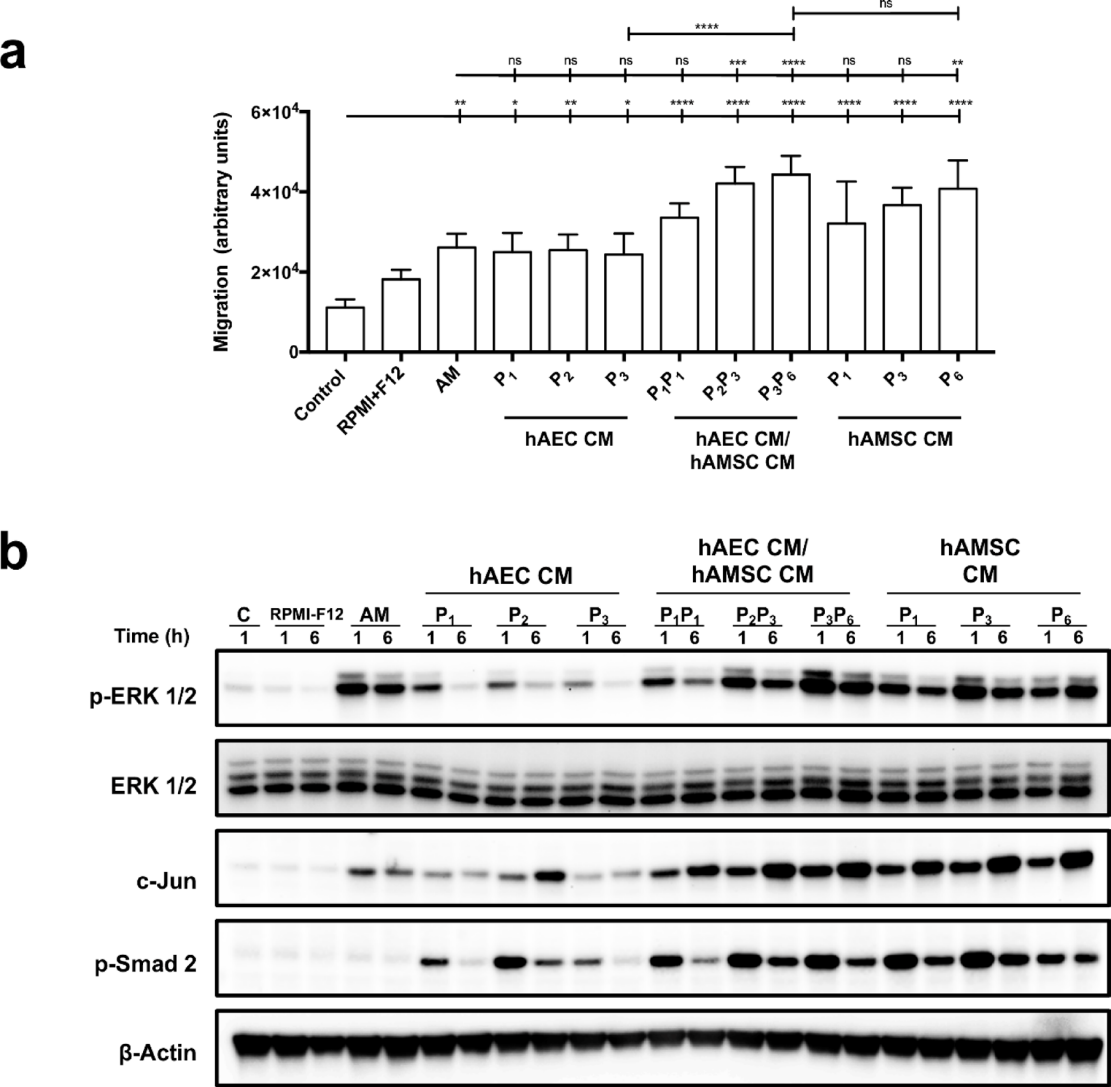
Mixes of conditioned media from hAEC and hAMSC stimulates the migration of serum starved HACaT cells and the phosphorylation of both ERK and c-Jun. (**a**) Confluent, serum-starved HaCaT cells (SS-HaCaT) were scratched and treated with hAM, hAEC-conditioned media (CM), hAMSC-CM, or a mix of CM. Three different CM from each cell source were tested, along with a mix of these media. RPMI-F12 served as the control for mixed media before conditioning. Data were derived from three independent experiments, with results presented as mean ± SEM. Asterisks denote statistically significant differences between conditions, as determined by one-way ANOVA followed by Tukey’s multiple comparisons test (ns: *p* > 0.05, *p < 0.05, **p < 0.01, ***p < 0.001, ****p < 0.0001). (B) Total protein extracts from subconfluent, serum-starved HaCaT cells treated with hAM, hAEC-conditioned media (CM), hAMSC-CM, or a mix of CM were analyzed. Three different conditioned media from each origin, along with their mix, were tested. Cells were stimulated for 1 and 6 h, and samples were analyzed by PAGE and western blot. RPMI-F12 was used as the control for mixed media before conditioning. Membranes were probed with antibodies against c-Jun, phospho-ERK1/2, and phospho-Smad2. β-actin was used as a loading control. *N* ≥ 3. A representative experiment is shown.

The results showed that the mixed CM were not more potent than hAMSC-CM or hAM alone in stimulating cell migration (Supplemental Fig. 2, Fig. 6a) or inducing c-Jun expression and ERK1/2 phosphorylation (Supplemental Fig. 3, Fig. 6b). These findings suggest that hAEC-CM contribute minimally to these phenomena, even when combined with hAMSC-CM.

Interestingly, the phosphorylation of Smad2 was significantly higher with both hAEC-CM and hAMSC-CM compared to hAM, where this effect was negligible (Fig. 6b).

Altogether, these data indicate that the effects of hAM on cell migration and the activation of migration-related proteins are primarily mediated by hAMSC, while hAEC play a limited role.

### hAMSC-CM and hAEC-CM produces the reorganization of focal adhesions related to cell migration

**Fig. 7:**
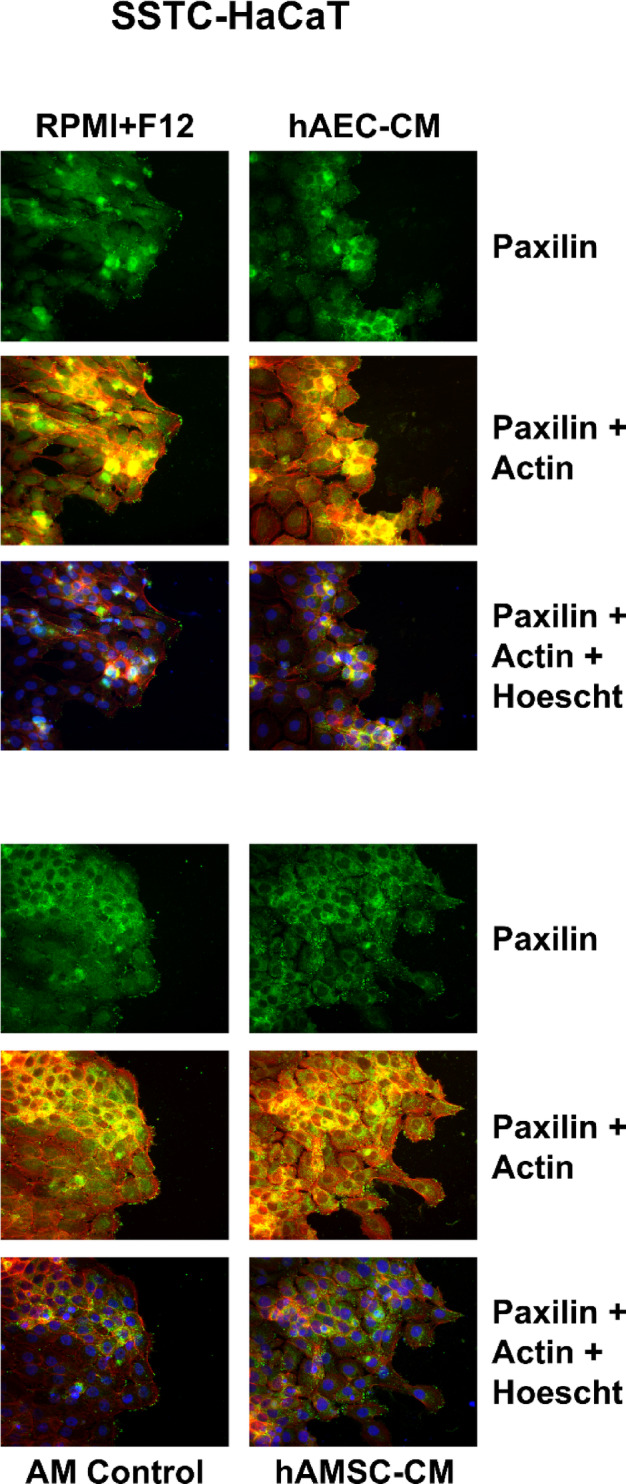
hAMSC conditioned media promotes migration through dynamization of cytoskeletal F-actin and FAs. Confluent SSTC-HaCaT (chronified) cells were scratched and allowed to migrate for 24 h. Cells were treated with either hAEC-CM, hAMSC-CM, hAM or a mixture of both media before conditioning (RPMI + F12). Then cells were immunostained with specific antibodies against paxillin (green), and co-stainied for phalloidin and Hoechst-33258 that were used to reveal actin cytoskeleton (red) and nuclei (blue). Representative images are shown. Scale bar indicates 50 μm. *N* ≥ 3.

We have observed that hAM effect on migration includes cytoskeleton and focal adhesion (FA) remodelling^25^. The effect of mesenchymal conditioned media on migration was considerably more potent that AM or hAEC´s CM (see Fig. 2a and b). Then, to assess the effect of the different CM on the migration front, we carried out immunocytochemistry assays in in vitro scratched SS- and SSTC-HaCaT cells targeting actin fibers (F-Actin) and paxillin. In SS-HaCaT, after 24 h stimulation with either different conditioned media or hAM, the simple apparency of the FA varied in the different samples. Unconditioned medium cells treatment show very few FA at the cell front, while hAM or hAEC-CM produced a moderate activation in the number of FA at the front edge of migration (Supplemental Fig. 4). Strikingly, the presence of hAMSC-CM produced a profusion of FA that were not only at the front edge of migration but in cells to the inside of the epithelium. When the same experiment was done in SSTC-HaCaT cells, the results were similar with a potentiation of FA production not only at the edge of migration but inside the epithelium (Fig. 7). Upon F-actin observation, the organization of actin was also modified in cells stimulated with either AM, hAEC-CM or hAMSC-CM in both SS-HaCaT and SSTC-HaCaT cells, with special attention at the formation of lamellipodia at the edge cells, that was more pronounced with the hAMSC-CM (Supplemental Fig. 4 and Fig. 7).

These data are coherent with the fact that, even though AM and hAEC-CM produced a positive migration of keratinocytes, the more powerful effect was seen with the hAMSC-CM.

### hAMSC-CM, but not hAEC-CM, rescues the cell cycle arrest induced by either serum deprivation or TGF-β treatment in HaCaT cells.

**Fig. 8:**
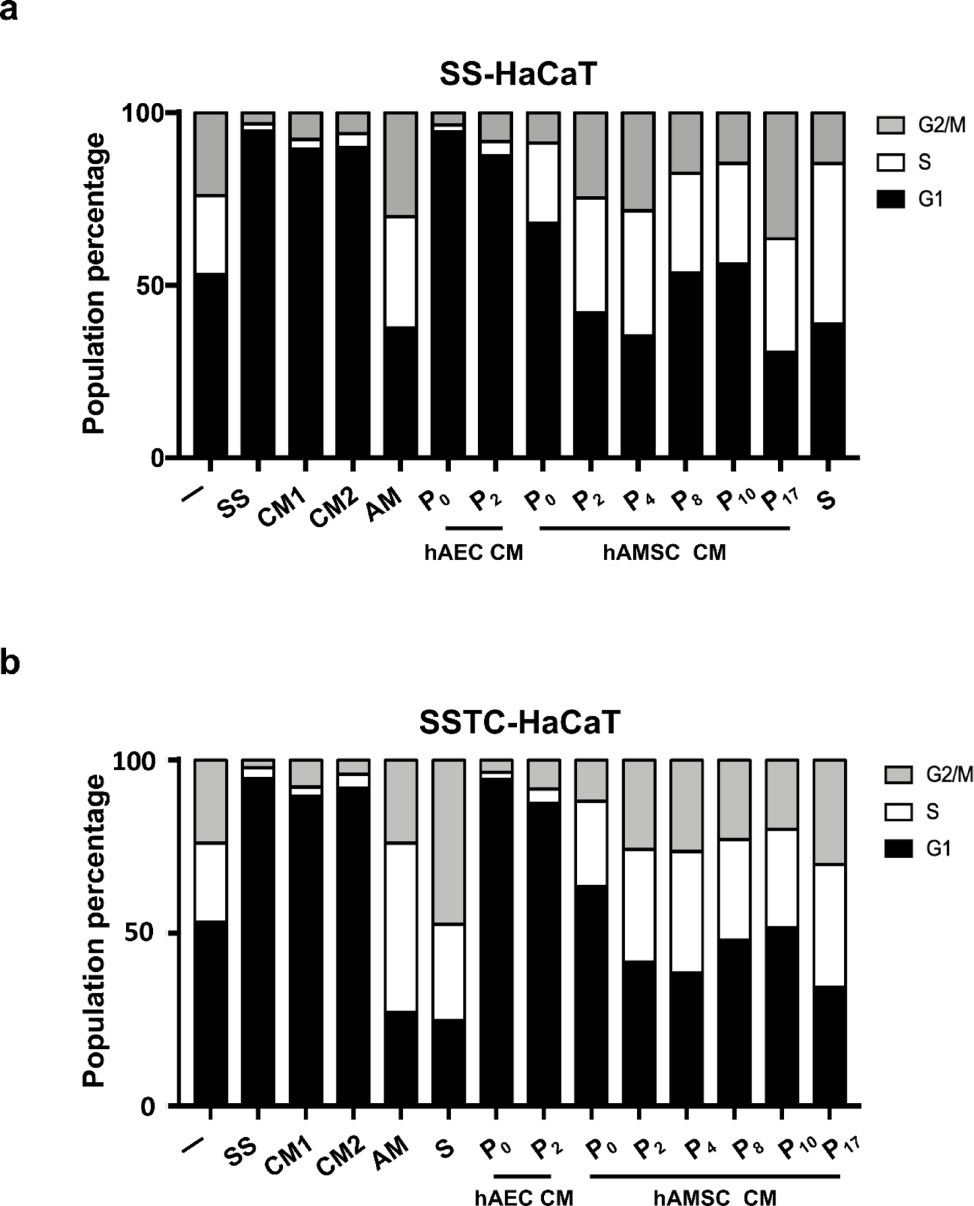
hAM treatment or conditioned medium from hAMSC restores proliferation in keratinocytes arrested by serum deprivation or SSTC-HaCaT cells persistently exposed to TGF-β. (**a**) Cell cycle progression was assessed by flow cytometry. Serum-starved HaCaT cells were treated with either hAM or conditioned media (CM) from epithelial or mesenchymal cells. HaCaT cells were analyzed at the indicated time points for each condition and treatment. (-) indicates cells grown in full serum medium without serum starvation treatment. SS refers to serum starvation treatment. CM1 represents unconditioned DMEM-F12 medium, while CM2 represents unconditioned RPMI1640 medium. CM from different cell passages are indicated. Data represent replicates from three independent experiments, with a single experiment shown. (**b**) Subconfluent SSTC-HaCaT cells were treated with either AM, serum starvation (SS), serum (S), or CM from hAEC or hAMSC cells. SSTC-HaCaT cells were analyzed at the indicated time points for each condition and treatment. (-) indicates no treatment. CM1 represents unconditioned DMEM-F12 medium, and CM2 represents unconditioned RPMI1640 medium. CM from different cell passages are indicated. Data represent replicates from three independent experiments, with a single experiment shown.

We have previously demonstrated that TGF-β treatment induced strong G1 cell cycle arrest can be effectively rescued by hAM^23^. hAMSC-CM and hAEC-CM cells were tested under 48-hour serum-starved conditions (SS-HaCaT). hAEC-CM failed to rescue the cells from serum deprivation, in contrast to hAM, which effectively restored cell viability. Notably, hAMSC-CM produced a robust rescue from cell cycle arrest, with this effect becoming evident from passage two onward (Fig. 8a).

TGF-β induces effective cell cycle arrest in keratinocytes, and when prolonged for 48 h (as in SSTC-HaCaT cells), it may also lead to a certain degree of inflammation, as indicated by increased IL-6 expression^34^. When SSTC-HaCaT cells were stimulated with hAM, they were relieved from cell cycle arrest. Interestingly, only hAMSC-CM replicated this effect, successfully rescuing SSTC-HaCaT cells from arrest (Fig. 8b).

These findings strongly suggest that hAMSC, but not hAEC, are responsible for hAM´s rescuing cells arrested by serum deprivation or TGF-β stimulation.

### CM derived from hAMSC attenuated the expression of genes associated with cell cycle arrest and inflammation induced by TGF-β

**Fig. 9:**
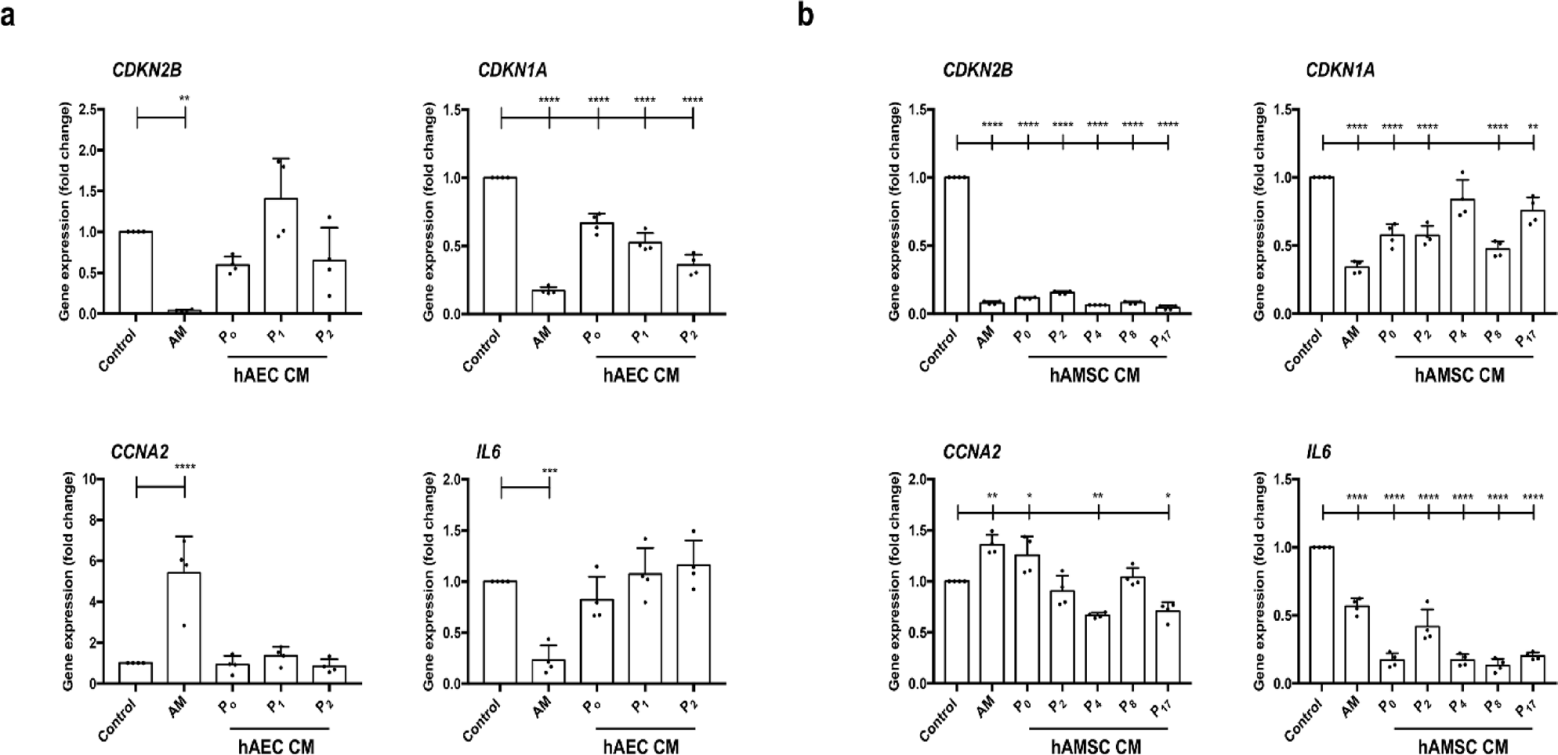
Amniotic membrane and hAMSC-conditioned media treatments improve the gene expression profile of SSTC-HaCaT cells persistently exposed to TGF-β. Genes related to the cell cycle (*CDKN2B*,* CDKN1A*,* CCNA2*) and inflammation (*IL6*) were analyzed. Samples were collected 24 h after treatment with the indicated media: (**a**) hAM or conditioned media (CM) from hAEC, and (**B**) hAM or CM from hAMSC. Gene expression levels are represented as fold change relative to the initial time point, with untreated cells maintained for the same duration as treated cells serving as the control. Data from three independent experiments were quantified by qPCR and are presented as mean ± SEM. Asterisks denote statistically significant differences between conditions, as determined by one-way ANOVA followed by Tukey’s multiple comparisons test (ns: *p* > 0.05, *p < 0.05, **p < 0.01, ***p < 0.001, ****p < 0.0001). *N* ≥ 3.

To investigate the potential gene regulation underlying the effects of hAMSC-CM, SSTC-HaCaT cells were treated for 24 h with hAM or CM derived from either hAEC or hAMSC. Key genes involved in cell cycle regulation and inflammation were analyzed. hAM treatment resulted in a significant change in the expression of p15 (*CDKN2B*) and p21 (*CDKN1A*). In contrast, hAEC-CM did not produce notable changes in either gene. (Fig. 9a). Furthermore, it failed to reverse the downregulation of Cyclin A2 (*CCNA2*), whereas hAM successfully restored its expression (Fig. 9a). Similarly, interleukin 6 (*IL6*), which was downregulated by hAM treatment, remained unchanged when hAEC-CM were applied (Fig. 9a).

A markedly different response was observed with hAMSC-CM. In general, the effects closely resembled those of hAM for all four genes tested (*CDKN2B*,* CDKN1A*,* CCNA2*, and *IL6*), with notable effects on *CDKN2B* and *IL6* (Fig. 9b).

These findings strongly indicate that hAMSC-CM, unlike hAEC-CM, can effectively rescue cells from the cell cycle arrest and inflammation induced by prolonged TGF-β treatment. Thus, hAMSC must be the cells responsible for hAM effect on these genes.

## Discussion

Recently, our lab has shown that the hAM is a powerful therapy for chronic wounds and diabetic foot ulcers^22,31,39,40^. In all cases, the application of the hAM resulted in remarkably successful outcomes for chronic and diabetic wounds. In this study, we aimed to evaluate the contribution of each cell population to the chronic wound-healing effects of the hAM. Cells were isolated following standard procedures and cultured in vitro to produce CM, that were tested for various wound-healing properties to determine the specific contributions of each cell type to these processes. Additionally, to investigate potential interactions between the two cell types, CM from epithelial and mesenchymal cells were mixed and analyzed.

Our findings clearly indicate that the effects observed in the mixed CM were entirely attributable to mesenchymal cells, both in terms of promoting migration and stimulating protein expression. This suggests that the therapeutic effects of hAM, as measured in our cell models, are primarily and most plausibly mediated by the mesenchymal cells present in the membrane.

The extraction of cells revealed distinct antigenic profiles, reflecting the different natures of the extracted cell populations. The epithelial cells exhibited a typical cuboidal shape and expressed epithelial markers. Additionally, the epithelial cells could be maintained in culture for approximately four passages before becoming senescent and losing their ability to proliferate. In contrast, hAMSC displayed a consistent mesenchymal phenotype that was sustained for at least 20 passages. It is important to note that usually, the extraction of mesenchymal cells is initially accompanied by minor epithelial contamination^41^. However, this contamination is quickly eliminated during cultivation in CHANG medium D©, a medium specifically designed to support mesenchymal cell growth^41,42^. Thus, after one passage, the presence of hAEC is drastically reduced, with hAMSC comprising more than 95% of the total cell population^41^. This shift may explain the relatively weak effect of CM obtained at passage 0 compared to the more pronounced effects observed with CM from higher passage numbers. Although reduced potency of early-passage hAMSC-CM may be partially explained by residual epithelial contamination according to our evidences, this remains a hypothesis that has not been fully confirmed. Ongoing proteomic analyses in our laboratory will help clarify these mechanisms. Accordingly, the interpretation of early- vs. late-passage CM potency should be considered under this limitation. Nevertheless, after that, the potency of the conditioned media was not related to the passage of the cells, showing similar results until passage 17 in all experimental set-ups. As a note, we acknowledge that the absence of fully matched passage comparisons between hAEC and hAMSC may bias interpretation. Therefore, our conclusions regarding differential potency between the two cell populations should be considered with this limitation in mind.

Migration is a key aspect of wound healing^43^. Studies have shown that the hAM accelerates keratinocyte migration from the wound edge and subsequently induces their differentiation, thereby contributing to the formation of an intact epithelium^44^. The effect of hAM on migration can be effectively monitored by the expression of c-Jun, which shows increasing levels of nuclear expression in cells at the leading edge of the wound^45^. Indeed, members of the AP-1 family had been involved in keratinocyte migration and wound healing process^29,46,47^. Also, migration enhancement can be measured by FA and F-actin cytoskeleton remodeling, that indeed are positively affected by AM stimulation^25^. Our initial tests with conditioned media revealed that, while media derived from epithelial cells were as effective as hAM in promoting cell migration, media from mesenchymal cells were significantly more potent. This was confirmed by FA and F-actin cytoskeleton remodelling. Altogether, this indicates the strong contribution of hAMSC to this phenomenon. However, the relatively lower potency of hAM itself may be attributed to the limited presence or reduced numbers of mesenchymal cells within the hAM matrix^37^, conditioned media lately extracted from hAMSC represented many more cells that the mesenchymal ones that would have been present at hAM when hAM is used at the experiments, multiplying the potency of the effect. Even with mixed media, the contribution of mesenchymal cells alone accounted for the effects observed in the combination of both media, including migration and protein stimulation. This indicates that the overall contribution of hAM to the effects measured in the cell models is most likely attributable to the mesenchymal cells present in the AM. Thus, hAM effect can be multiplied when more hAMSC contribute to the CM.

We recognize that β-actin exposure varied in some western blot experiments, and that cross-blot comparisons may introduce inconsistencies. To minimize this issue, only same-gel analyses were quantified. Wounds treated with hAM exhibit a strong induction of c-Jun expression, both at the wound edge in wound healing scratch assays using HaCaT cells and in human wounds^23^. When tested on HaCaT cells, the presence of either CM induced migration that was consistent with the overexpression of c-Jun. Surprisingly, the stronger migratory effect observed with hAMSC-CM was accompanied by a more pronounced overexpression of c-Jun at the protein level, along with increased and more robust expression of c-Jun at the leading edge of the wound in HaCaT scratch assays. This suggests that the effect of hAM on cell migration is primarily due to the involvement of mesenchymal cells. Notably, the mixture of both epithelial and mesenchymal CM neither enhanced nor diminished the effect of mesenchymal CM on migration.

hAEC-CM has been shown to elicit certain wound healing-related parameters, such as promoting migration and increasing the proliferation of keratinocytes^48^. When CM were evaluated for their effect on proliferation, only hAMSC CM were able to relieve the cell cycle arrest induced by serum deprivation. This suggests that certain factors released by mesenchymal cells are critical for overcoming cell arrest imposed by the lack of growth factors. The Ras/Raf/MEK/ERK signaling cascade plays a central role in integrating a wide range of extracellular stimuli into key biological responses, regulating processes such as cell proliferation, differentiation, and apoptosis^49^. ERK activation is required for cell proliferation^50^. Indeed, the lack of serum results in a reduction in ERK phosphorylation, which correlates with cell cycle arrest. This arrest is relieved by the presence of AM, which also induces the phosphorylation of ERK, facilitating cell cycle progression^23,34^. The ERK phosphorylation response in serum-starved HaCaT cells to hAEC-CM was much weaker compared to hAM. In contrast, the phosphorylation of ERK1 and ERK2 was significantly stronger with hAMSC-CM than that with hAM, which was in clear coherence with the release from cell cycle arrest experienced by serum-deprived HaCaT cells. Furthermore, the mixture of both hAMSC-CM and hAEC-CM exhibited properties similar to those of hAMSC-CM alone. The future analysis and comparison of the components of both conditioned media would undoubtably clarify which components are necessary to exert this pro-proliferative effect.

TGF-β produces a strong G1 arrest when applied to epithelial cells, such as HaCaT cells^51^. When applied persistently to HaCaT cells in the absence of serum, TGF-β induces changes in cell morphology, causing the cells to adopt a more spread-out shape. Additionally, TGF-β alters some of their epithelial characteristics, reflecting the onset of EMT^34^. Moreover, the SSTC-HaCaT cells generated in this manner exhibit strong expression of antiproliferative genes such as *CDKN2B* (p15) and *CDKN1A* (p21), along with the downregulation of the pro-proliferative gene *CCNA2* (cyclin A2)^34^. While the presence of hAEC-CM did not affect the expression of these genes, both AM- and hAMSC-CM induced their strong downregulation. This is consistent with the resumption of cell cycle activity observed after treating cells with hAMSC-CM. When CM from both hAEC and hAMSC were tested for Smad2 phosphorylation, a positive response was observed in both types of cells. hAEC are known to produce TGF-β upon isolation, which may drive their own EMT^38^. Because TGF-β causes a strong cell cycle arrest at G1^52^, it could be speculated that the inefficacy of hAEC to produce the same benefits as hAM could be due to their TGF-β increased production after isolation^38^. However, it is difficult to consider this as a possibility, given that the expression of TGF-β is expected to be more pronounced in hAMSC cells isolated from hAM, owing to the ability of hAMSC-conditioned media to induce the phosphorylation of Smad2. Despite this, these cells can overcome cell-cycle arrest and transition into proliferation, not only under serum starvation conditions but also in response to TGF-β chronification or stimulation. Furthermore, this aligns with the need for an attenuated but functional TGF-β signaling pathway, which is essential for the cell migration stimulated by hAM^26,27^. Further research is needed to clarify this interesting feature of hAMSC.

*CDKN2B* is a potential effector of TGF-β-induced cell cycle arrest that exert its effect through the inhibition of CDK4 and CDK6 kinases^53^. TGF-β is believed to induce G1 cell cycle arrest by transcriptionally upregulating CDK inhibitors, such as *CDKN2B* (p15) and, in some cell types, *CDKN1A* (p21), while simultaneously downregulating the CDK-activating phosphatase *CDC25A*^52^. In the absence of *CDKN2B*,* CDKN1A* appears to compensate and fulfill its role^54^. Interestingly, the hAM has been shown to downregulate the expression of both *CDKN2B* and *CDKN1A*, even in the presence of TGF-β. This suggests a paracrine mechanism mediated by hAM, potentially involving activation of the MAPK/Erk pathway^23] , [26^. Consequently, hAMSC may be responsible for the pro-proliferative effect exerted by hAM, even under conditions of TGF-β stimulation.

In SSTC-HaCaT cells, chronic stimulation with TGF-β results in the downregulation of the pro-proliferative gene *CCNA2* (Cyclin A2). However, the presence of hAM is able to reverse this downregulation^33,34^. Similarly, hAMSC-CM—but not hAEC-CM—was able to upregulate back *CCNA2* (Cyclin A2). The specific functions of cyclin A protein at different stages of the cell cycle depend on its CDK partners. Cyclin A is crucial for at least two critical points in the somatic cell cycle: during the S phase, through the activation of CDK2, and during the G2-to-M transition, through the activation of CDK1^55,56^. The transcriptional regulation of *CCNA2* (cyclin A2) is influenced by peripheral signals such as growth factors, TGF-β, and cell interactions with the ECM^57,58^. Moreover, there is a positive correlation between *CCNA2* upregulation and increased cell migration^59^. Therefore, the ability of hAMSC CM to restore *CCNA2* (Cyclin A2) expression may have a dual role: facilitating cell cycle progression and enhancing cell migration—two undeniably beneficial features for wound healing.

The proliferation phase of healing is intricately regulated by inflammation, with IL-6 playing a particularly significant role in this process^60^. IL-6 can be detected in the blood during chronic wound progression or in patients with other pathologies associated with delayed wound healing^61^. Prolonged inflammation disrupts granulation tissue formation, potentially leading to chronic wounds or hypertrophic/keloid scars^62^. Therefore, the regulation of *IL-6* levels in SSTC-HaCaT cells by hAM aligns with the known role of hAM in managing chronic wounds^33,34^. Of the two CM tested—hAEC-CM and hAMSC-CM—only hAMSC-CM was able to downregulate *IL-6* expression in SSTC-HaCaT cells. This finding is consistent with the observed effects of hAM on chronic wounds and suggests the significant therapeutic potential of the hAMSC-CM in treating such wounds. hAMSC have attracted much attention due to their immunomodulatory properties^63^ and also due to their paracrine actions and prospective applications in regenerative medicine^63^. Compared to hAEC, the paracrine effect of hAMSC has been shown to be more potent in terms of immunomodulation^41^. Similarly, in our study, the paracrine effect of hAMSC on cell migration surpassed that of the hAM. This was also evident in the expression of proteins related to migration. However, the effect of hAEC on most of the parameters measured, apart from migration, was negligible.

Future research has to determine the specific composition of either conditioned medium that give it the specific features described in this paper, and compare to the composition of the conditioned media coming from the incubation of AM alone. Moreover, further confirmation of this findings could be done in animal models deficient for wound healing, future research is needed to asses this.

Finally, the future challenge lies in harnessing the benefits of hAMSC to treat chronic wounds and ulcers, enabling many patients suffering from these conditions to benefit from such therapies. Further technological advancements will be required to translate these promising effects into effective and reliable treatments for this type of wound, however the present results are positive and encouraging to commence this road.

## Methods

### Isolation and characterization of hAMSC and hAEC

Placentas were obtained from cesarean section with previous patient informed-consent procedure. This procedure is approved by the *Hospital Clínico Universitario Virgen de la Arrixaca Ethical Committe*. Human amniotic membrane (hAM) was peeled off mechanically from the underlying chorion and washed in physiological saline solution (PSS) supplemented with cotrymazol (48 µg/mL) (Almirall-Prodefarma S.A., Barcelona, Spain), tobramycin (50 µg/mL) (Laboratorios Normon S.A., Madrid, Spain), and vancomycin (50 µg/mL) (Laboratorios Hospira S.L., Madrid, Spain). Purification of hAEC and hAMSC were isolated as previously described in Miki et al.. 2006 and Magatti et al.. ^42,46^ and Magatti et al.. 2016^42^ respectively. Briefly, hAEC were obtained by trypsin digestion with 0.05% trypsin/EDTA solution (Sigma, Madrid, Spain) whereas hAMSC were digested with collagenase A (Sigma, Madrid, Spain). Characterization of the hAEC and hAMSC populations was carried out using flow cytometry. The cells were analyzed for the expression of markers including CD73, CD90, CD105, CD13, CD66, E-Cadherin, and SSEA-4. hAEC were seeded at a density of 100.000 cells/cm^2^ in DMEM-F12 Hepes (Biowest, Nuaillé, France) supplemented with 10% fetal bovine serum (FBS) (Thermo Fisher Scientific, Rockford, IL, USA), 10 ng/mL epidermal growth factor (EGF, Sigma-Aldrich, St. Louis, MO, USA), 1% L-glutamine, and 1% penicillin/streptomycin (both from Biowest, Nuaillé, France) (complete DMEM-F12 Hepes medium), and incubated in a humidified atmosphere at 37 °C with 7.5% CO_2_ following manufacturer recommendation. hAMSC were seeded at a density of 10.000 cells/cm^2^ in CHANG medium D© (FUJIFILM, Barcelona, Spain) 1% penicillin/streptomycin (Biowest, Nuaillé, France) and incubated in a humidified atmosphere at 37 °C with 5% CO_2_ following manufacturer recommendation. Note that an epithelial cells contamination at the hAMSC is unavoidable, however their culture in CHANG medium D© produced its selective elimination in the first passage^42^. Images of the different cell passages were taken with a ZEISS Axiovert 5 coupled with an Axiocam 208 Color.

### Conditioned media preparation

For epithelial cells, the medium used for conditioning was the same epithelial growth medium, but with serum removed and still supplemented with 1% L-glutamine, and 1% penicillin/streptomycin. However, for hAMSC conditioning, CHANG medium D© could not be used due to its complex composition, which includes several growth factors and hormones. Instead, an alternative serum-free medium was employed to ensure reliable comparisons. In this case, we used the medium employed during cell purification RPMI1640 (Sigma-Aldrich, Darmstadt, Germany) FBS deprived supplemented with 1% L-glutamine, and 1% penicillin/streptomycin. The procedure was standardized to maintain a consistent ratio of cells to the volume of media across different lots and between mesenchymal and epithelial cells. Briefly, Conditioned Media (CM) was obtained from hAEC by culturing the cells for 48 to 65 h in complete FBS and EGF deprived DMEM/F12-Hepes at 37 °C and 7.5% CO₂, yielding conditioned media equivalent to 2.5 to 5 × 10⁵ cells per milliliter. Similarly, CM was obtained from hAMSC by culturing the cells for 48 to 65 h in serum-deprived RPMI1640 (Sigma-Aldrich, Darmstadt, Germany) supplemented with 1% L-glutamine, and 1% penicillin/streptomycin at 37 °C and 5% CO₂, also yielding media equivalent to 2.5 to 5 × 10⁵ cells per milliliter. Once either CM was collected, it was centrifuged at 4,000 rpm for 10 min at 4 °C. Cell debris was discarded, and the supernatant was collected and stored at -80 °C for future use. In all experiments, either DMEN/F12 Hepes or RPMI1640 pre-conditioned media were used as controls. To clarify further, in this study, conditioned medium inputs were equalized by cells/volume, rather than by absolute cell number present in hAM, to ensure comparability across groups. This approach deviates from the physiological epithelial-to-mesenchymal ratio in the native hAM; however, similar strategies have been widely adopted in CM-based studies to allow functional assessment under standardized conditions^41^.

### HaCaT cell culture and TGF-β chronification of HaCaT cells

HaCaT cells, a human spontaneously immortalized keratinocyte cell line, was obtained from Dr. Caroline S. Hill (London Research Institute, Cancer Research, UK). The cells were cultured in Dulbecco’s Modified Eagle Medium (DMEM) high-glucose (Biowest, Nuaillé, France), supplemented with 10% fetal bovine serum (FBS) (Thermo Fisher Scientific, Rockford, IL, USA), 1% L-glutamine, and 1% penicillin/streptomycin (both from Biowest, Nuaillé, France) at 37 °C in a humidified atmosphere with 7.5% CO₂, following manufacturer instructions.

The chronification of HaCaT cells was carried out as follows: cells were seeded in 6 cm culture plates to reach 50% confluence. They were then stimulated with TGF-β (2 ng/mL) (PrepoTech, Rocky Hill, NJ, USA) in serum-free growth media for 24 h. After this initial stimulation, the same dosage of TGF-β was applied again for an additional 24 h. This process produced Serum Starved TGF-β Chronified (SSTC)-HaCaT cells, as described previously^33,34^. In parallel, as a control, HaCaT cells were cultured under serum-starved conditions for 48 h without TGF-β treatment, resulting in Serum Starved (SS)-HaCaT cells. To test conditioned media, half of the culture medium was replaced with CM from either hAEC or hAMSC, supplemented with TGF-β (2 ng/mL) (PrepoTech, Rocky Hill, NJ, USA) adjusted for the new volume, and incubated for 24 h.

### Western blot

HaCaT cells were grown on 6 cm culture plates until they reached 50–60% confluence. Then, cells were serum starved and after 24 h, they were stimulated with hAM or conditioned media derived from either hAEC or hAMSC in the presence TGF-β (2 ng/mL) (PrepoTech, Rocky Hill, NJ, USA) for the indicated times. For protein extraction, cells were rinsed twice with PBS and lysed with 20 mM HCl-Tris pH = 7.5, 150 mM NaCl, 1 mM EDTA, 1.2 mM MgCl_2_, 0.5% Nonidet p-40, 1 mM DTT, 25 mM NaF, β-glycerophosphate supplemented with phosphatase (I and II) and protease inhibitors (all from Sigma Aldrich, St Louis, MO, USA). Protein content in the lysates was quantified and normalized using the Bradford method. The protein extracts were then analyzed by SDS-PAGE, followed by western blotting with the appropriate antibodies. Images of the blots were captured for analysis. Images were taken with a Chemidoc XRS1 (Bio-Rad, Hercules, Ca, USA). Western blot bands were quantified using BioRad Image Lab 6 software. For all Western blot analyses, densitometric quantifications were derived exclusively from samples run on the same gel/blot to avoid cross-blot variability. The experiment was done at least three times. A representative image is provided in any case for illustrative purposes.

### Wound healing scratch assay

Wound healing scratch assays were performed as described previously^65^. Briefly, HaCaT cells were grown in 24-well plates until reaching 100% confluence, after which they were serum-starved for 24 h. A cross-shaped scratch was made on the cell layer using a sterile 40 µL micropipette tip. The culture medium in which the cells were grown was set aside for later re-incorporation. The cells were then washed with FBS-free culture medium to remove unattached cells. The original medium was centrifuged to eliminate debris and floating cells and was subsequently returned to the scratched HaCaT cells.

Immediately after, the cultures were treated with either 1 cm x 1 cm hAM fragments floating on the culture medium or CM derived from hAEC and hAMSC, which were added in a 1:1 ratio to the serum-starved medium. To assess migration, images of the artificial wounds were captured at the start of the experiment (time 0). After 24 h, the cells were fixed with 4% formaldehyde in PBS, washed twice with PBS, and new images of the wounds were captured. Images were acquired using a ZEISS Axiovert 5 microscope equipped with an Axiocam 208 Color camera at 10× magnification.

The areas before and after cell migration were measured using ImageJ Fiji Software. Briefly, the wound area was calculated by subtracting the area remaining after migration from the original wound area^66^. Theses experiments were done at least three times. A representative image is provided in any case for illustrative purposes.

### Cell cycle analysis

Firstly, HaCaT cells, converted into either SS-HaCaT or SSTC-HaCaT (as described above) were stimulated with CM [half replacement of the culture medium, supplemented with additional TGF-β (2 ng/mL) when required] or hAM for 24 h. Then cells were trypsinized and fixed with 70% ice-cold ethanol and phosphate buffered saline (PBS) for 30 min. Afterwards, the cells were centrifuged at 1000 rpm for 5 min. Cells were treated with 20 µg /mL of RibonucleaseA (Sigma-Aldrich, St Louis, MO, USA) solution and stained with 400 µg/ml propidium iodide (Sigma-Aldrich, St Louis, MO, USA). Cells were analyzed by flow cytometry using a LSRFortessa X-20 (BD sciences, Pharmigen, Beckton Dickinson, Franklin Lakes, NJ, USA). The experiment was done at least three times. 

### Gene expression

For gene analysis, total RNA (800 ng) was retro-transcribed using iScript reagents (Bio-Rad, Hercules, CA, USA). The resulting cDNA was utilized for qPCR using SYBR premix ex Taq (Takara Bio Europe/Clontech, Saint-Germain-en-Laye, France). Gene expression levels were normalized to the glyceraldehyde 3-phosphate dehydrogenase (*GADPH*) content by applying the Cq method (2^−∆∆Cq^). Replicates from at lest three independent experiments were quantified and the results are respresented. Analyzed data represent mean ± SEM. A list of primers used for gene expression analysis is provided in Table 2.

**Table 2:** Primers used for gene expression analysis by qPCR.

Primer name	Primer sequence 5′ to 3′
*GAPDH*-Fwd	ACCACAGTCCATGCCATCAC
*GAPDH*-Rev	TCCACCACCCTGTTGCTGTA
*CDKN2B*-Fwd *CDKN2B*-Rev	ATGCGCGAGGAGAACAAG CTCCCGAAACGGTTGACTC
*CDKN1A*-Fwd	ATGTCAGAACCGGCTGGGGATG
*CDKN1A*-Rev	GGGCTTCCTCTTGGAGAAGATC
*IL6*	Proprietary sequence (Qiagen QuantiTect)
*CCNA2*	Proprietary sequence (Sigma KiCqStart)

### Inmunofluorescence

Immunostaining was performed as previously described^38^. Briefly, either hAEC or hAMSC were seeded on 6 cm culture plates over round glass coverslips and left to grow. At the indicated times, cells were fixed with 4% formaldehyde in PBS at room temperature. Subsequently, they were permeabilized in 0.3% Triton X-100 in PBS for 15 min. Then, coverslips were incubated with blocking buffer containing 0.3% bovine serum albumin, 10% FBS, 0.1% Triton X-100 in PBS supplemented with 5% skimmed milk for 30 min. Samples were incubated for 1 h with an appropriate primary antibody diluted in blocking buffer deprived of skimmed milk. Following primary antibody incubation, the coverslips were washed and subsequently incubated with the secondary antibody in the same blocking buffer, along with Hoechst 33,258 (Fluka, Biochemika, Sigma-Aldrich, St. Louis, MO, USA) to stain the nuclei. The coverslips were mounted and prepared for observation. Images were taken with a confocal microscope (LSM 510 from ZEISS, Jena, Germany). The experiment was done at least three times. Representative image are provided for illustrative purpouses.

### Antibodies

Primary antibodies used included anti-panCytokeratin, anti-E-cadherin, paxillin and anti-vimentin (all from Santa Cruz Biotechnology, Heidelberg, Germany); anti-alpha smooth muscle actin (α-SMA) and anti-β-actin (both from Sigma-Aldrich, St. Louis, MO, USA); anti-phospho-ERK1/2, anti-ERK1/2, anti-c-Jun, and anti-p-Smad2 (all from Cell Signaling Technology, Danvers, MA, USA); and anti-Smad2/3 (Becton Dickinson, Franklin Lakes, NJ, USA).

Secondary antibodies included Alexa Fluor 488-labeled goat anti-mouse (Molecular Probes, Thermo Fisher Scientific, Waltham, MA, USA) and horseradish peroxidase (HRP)-linked anti-rabbit IgG F(ab’)₂ fragment (from donkey) (GE Healthcare, Little Chalfont, United Kingdom). To stain nuclei and actin, Hoechst 33,258 (Fluka, Biochemika, Sigma-Aldrich, St. Louis, MO, USA) and Phalloidin CruzFluor™ 594 Conjugate (Santa Cruz Biotechnology, Heidelberg, Germany) were used.

### Cell-front migration assay, subcellular localization assay by immunofluorescence

HaCaT cells were cultured on round glass coverslips placed in 6 cm diameter plates containing complete DMEM medium until reaching 100% confluence. At this point, the cells were washed and subjected to a 24 + 24-hour serum starvation, either in the absence or presence of TGF-β. Following serum starvation, the epithelium of SS-HaCaT or SSTC-HaCaT cells was scratched using a razor blade to create a gap large enough to monitor cell migration over 24 h. The cells were then returned to culture plates under their original growth conditions. The newly created wound was designated as time 0 of the experiment. To stimulate the wounded cells, hAM in FBS-free DMEM was placed over the cells, or different conditioned media were added at a 1:1 ratio. Typically, 24 h after wounding, the glass coverslips were fixed with 4% formaldehyde in PBS for 10 min and washed twice with PBS. The cells were then immunostained following the previously described immunostaining protocol (see above). After immunostaining, image acquisition was performed using a confocal microscope (LSM 510 META, ZEISS, Jena, Germany). The images were processed using the Zeiss Efficient Navigation (ZEN) interface software (ZEISS, Jena, Germany). For a broader view of the migration front, particularly for c-Jun staining (as indicated in the Figures), 4 × 3 linked fields were captured using the “Tile Scan” tool in the ZEN software. For the quantification of c-Jun levels in immunofluorescence, images were analyzed and quantified by Image J software as described in Stelling Férez et al., 2023^67^. Briefly, 8-bit, three-channel (Red, Green, Blue, RGB) images were separated into three individual color channels, resulting in three monochromatic images. The blue channel (Hoechst staining) was used to identify nuclei, and Regions of Interest (ROIs) were established for each nucleus, creating an ROI mask for every nucleus in the image. These ROI masks were then overlaid onto the corresponding green channel image (c-Jun staining) to calculate the green intensity value for each nucleus (ROI). Due to the large area covered in each image (Tile Scan), the pictures were divided into four equal sectors (S1, S2, S3, and S4), with S1 representing the outermost edge of the wound. Within each sector, the quantified intensity values of individual nuclei were treated as replicates to obtain c-Jun intensity data under the respective experimental conditions. The experiment was done at least three times. A representative image is provided for illustrative purpouses.

### Statistical analysis

Data are expressed as mean ± SD (standard deviation). All statistical analyses were performed using GraphPad Prism 7 software. A p-value less than 0.05 was considered statistically significant. In figure legends, asterisks indicate statistically significant differences between conditions as follows: ns (not significant, *p* > 0.05), *p < 0.05, **p < 0.01, ***p < 0.001, and ****p < 0.0001.

Differences between the means of different groups were determined using a one-way ANOVA test. To compare differences between individual pairs of means, Tukey’s post hoc test was performed.

## Supplementary Information

Below is the link to the electronic supplementary material.

Supplementary Material 1

Supplementary Material 2

## Data Availability

Data is provided within the manuscript or supplementary information files.

## Declarations

The authors declare that they have not used AI-generated work in this manuscript.
