# Supplementary material for: Distinct roles of amniotic membrane epithelial (hAEC) and mesenchymal stromal cells (hAMSC) in amniotic membrane-driven wound healing
Source: Sci Rep. 2025 Oct 21;15:36806. doi: 10.1038/s41598-025-20685-4 (PMC12540749; doi:10.1038/s41598-025-20685-4)
Supplement: Supplementary file 1 — Supplementary Material 1 [file 41598_2025_20685_MOESM1_ESM.pdf]

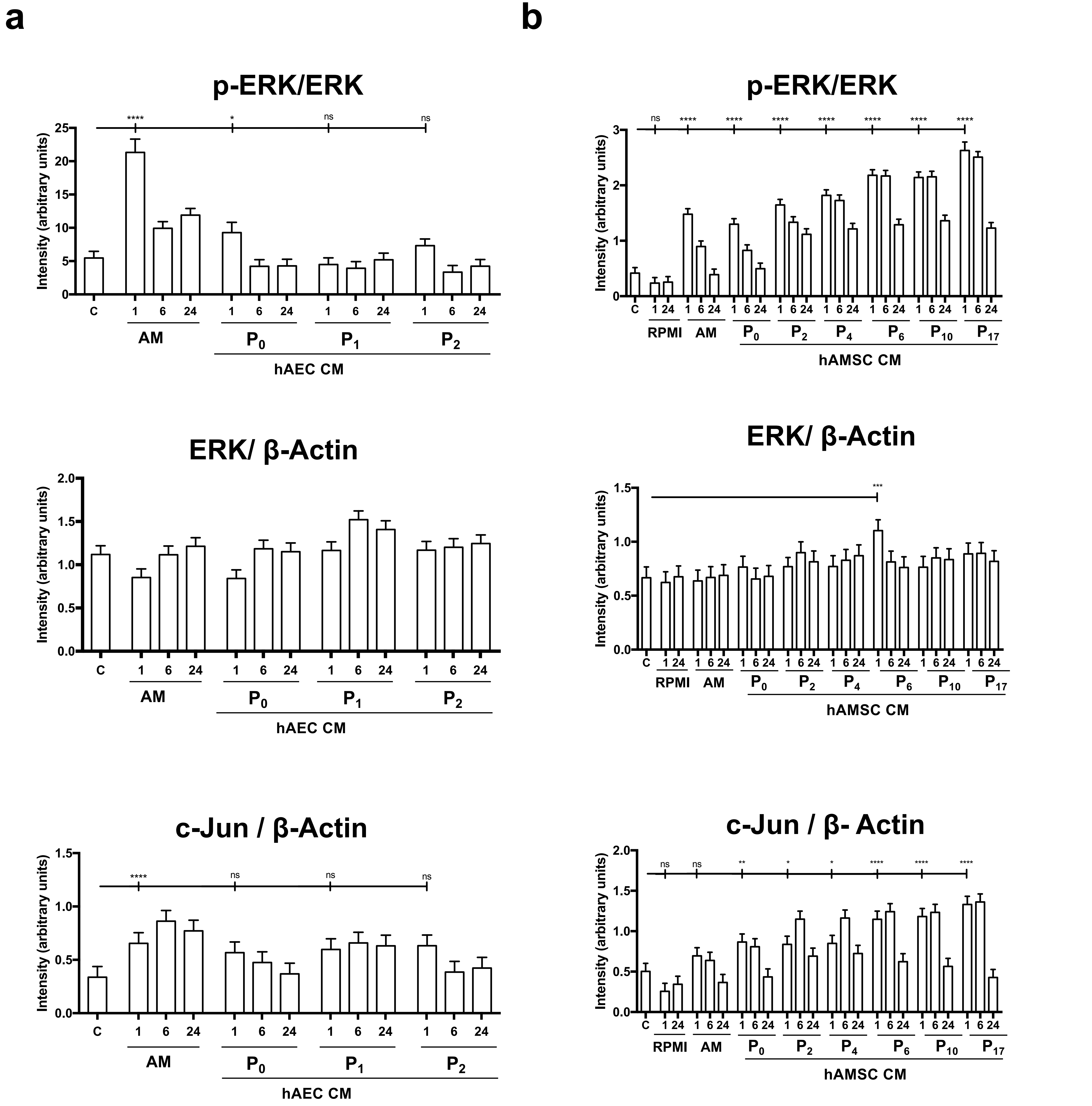
a
b
p-ERK/ERK
Intensity (arbitrary units)
****
*
ns
ns
C
1
6
24
AM
P_0
P_1
P_2
hAEC CM
ERK/ β-Actin
c-Jun / β-Actin
p-ERK/ERK
RPMI
P_2
P_4
P_6
P_{10}
P_{17}
hAMSC CM
ERK/ β-Actin
***
c-Jun / β- Actin
**

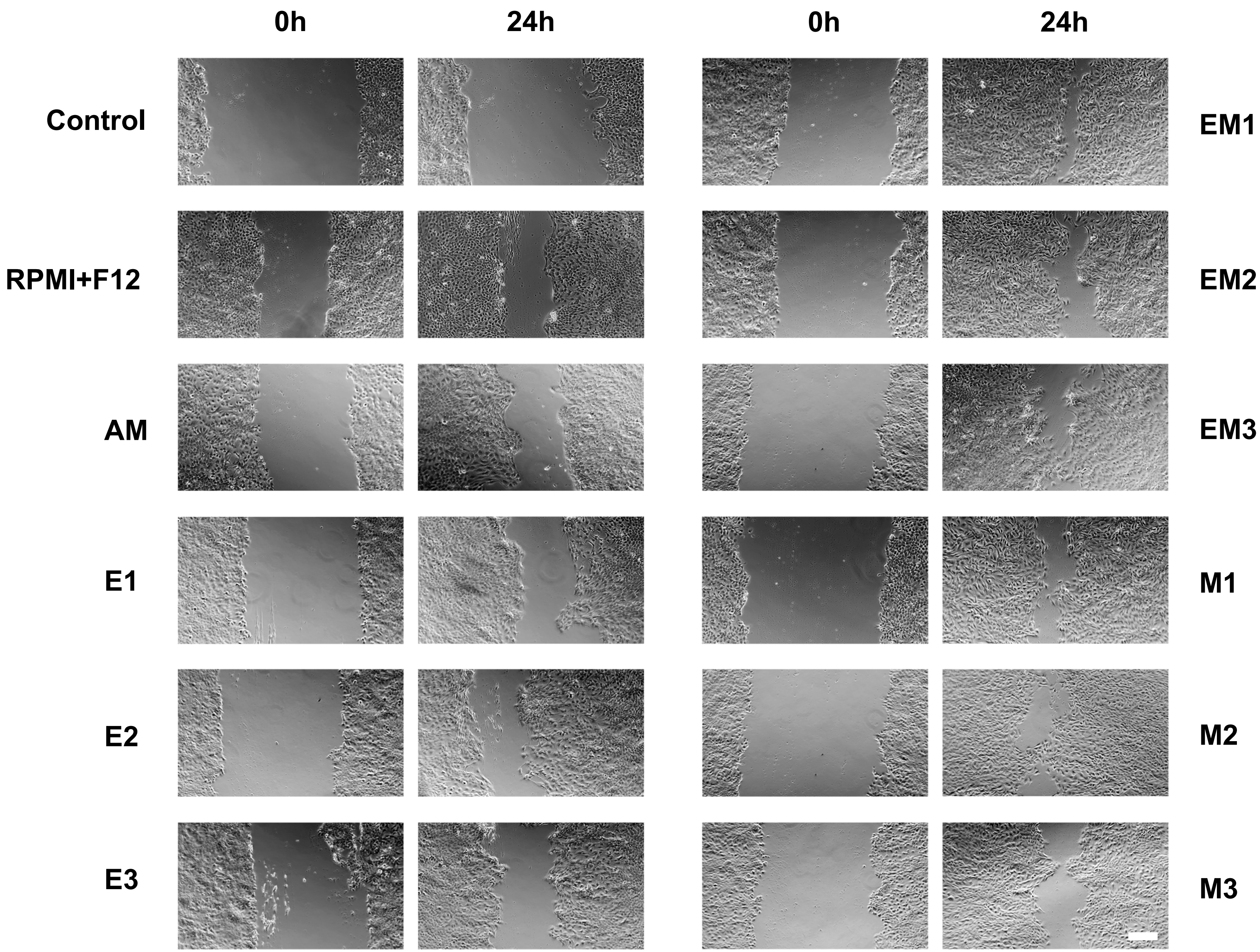
0h
24h
0h
24h
Control
RPMI+F12
AM
E1
E2
E3
EM1
EM2
EM3
M1
M2
M3

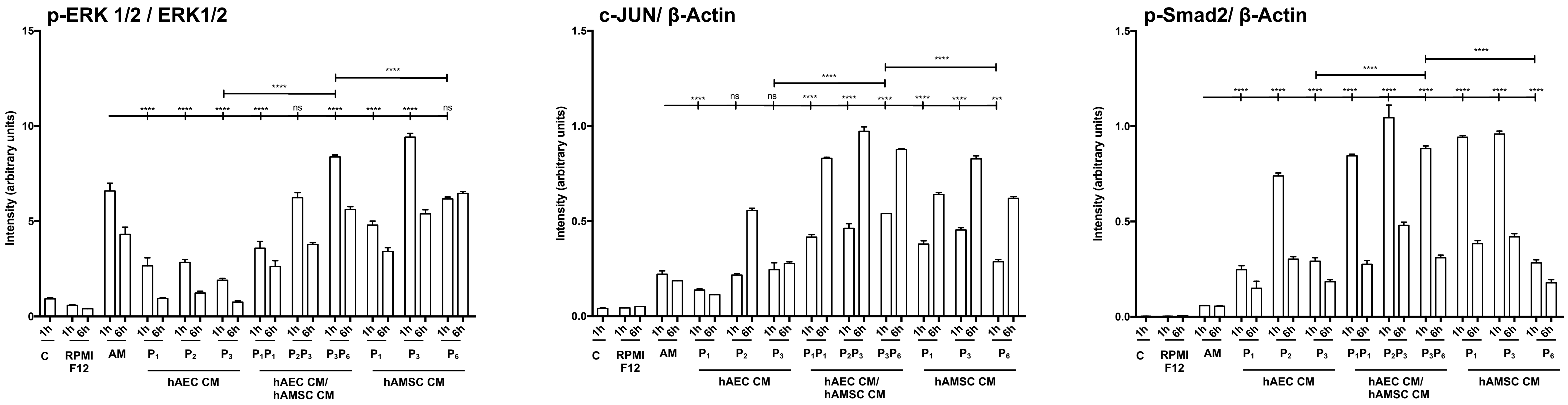

p-ERK 1/2 / ERK1/2
c-JUN/ β-Actin
p-Smad2/ β-Actin
Intensity (arbitrary units)
0
5
10
15
0.0
0.5
1.0
1.5
****
ns
***
1h
6h
C
RPMI
F12
AM
P1
P2
P3
P1P1
P2P3
P3P6
P6
hAEC CM
hAEC CM/
hAMSC CM
hAMSC CM

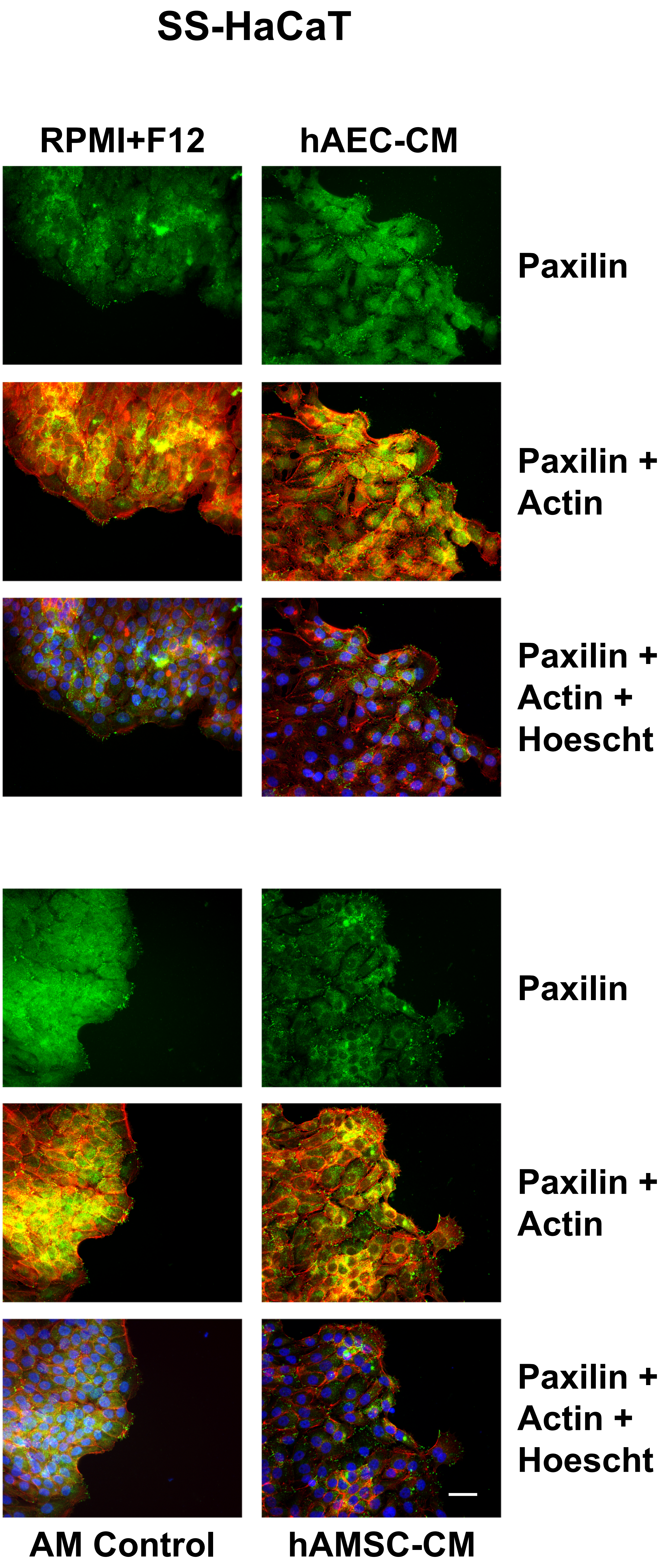
SS-HaCaT
RPMI+F12
hAEC-CM
Paxilin
Paxilin + Actin
Paxilin + Actin + Hoescht
Paxilin
Paxilin + Actin
Paxilin + Actin + Hoescht
AM Control
hAMSC-CM

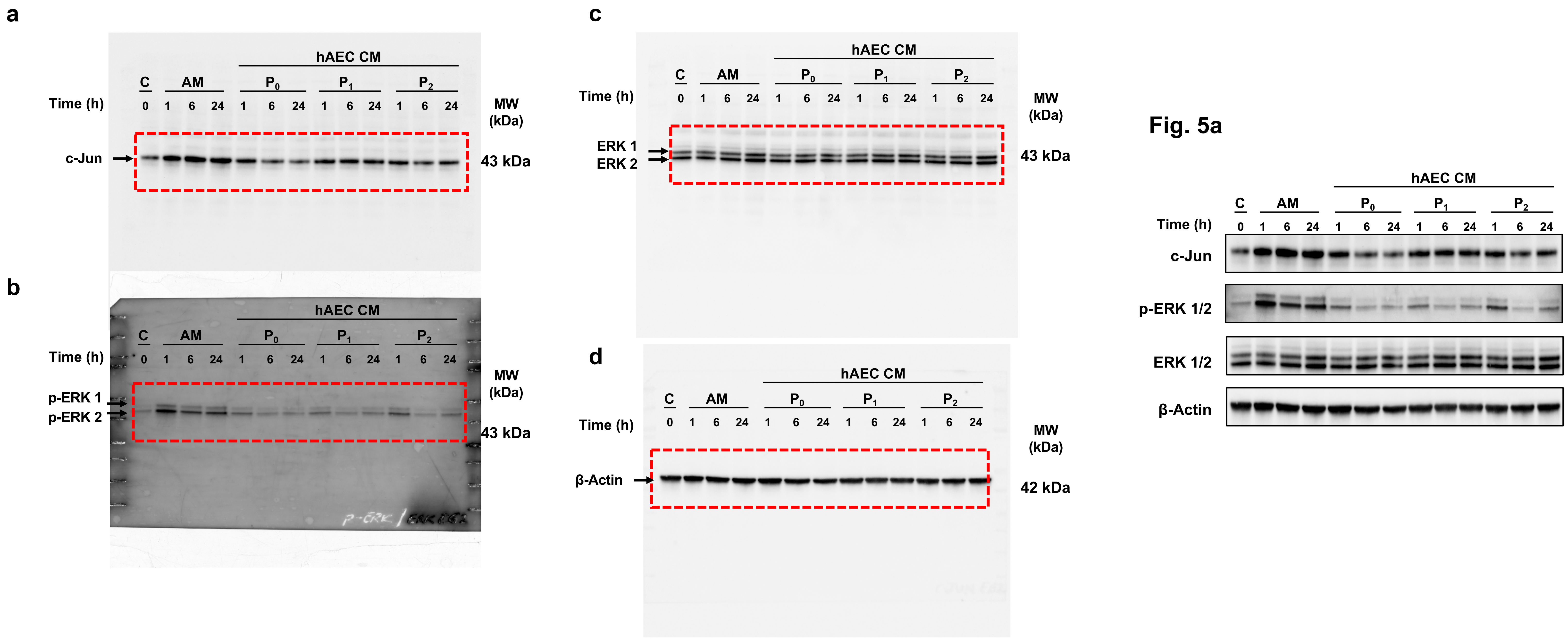

Supplemental Figure 3. Full-length blots corresponding to crops showed in Figure 5a (a) c-Jun (b) Thr 202/Tyr 204 Phosphorylated ERK (c) ERK1/2 (d) Beta-actin loading. Dashed red rectangle indicates the portion of the blot that was used in the figure.

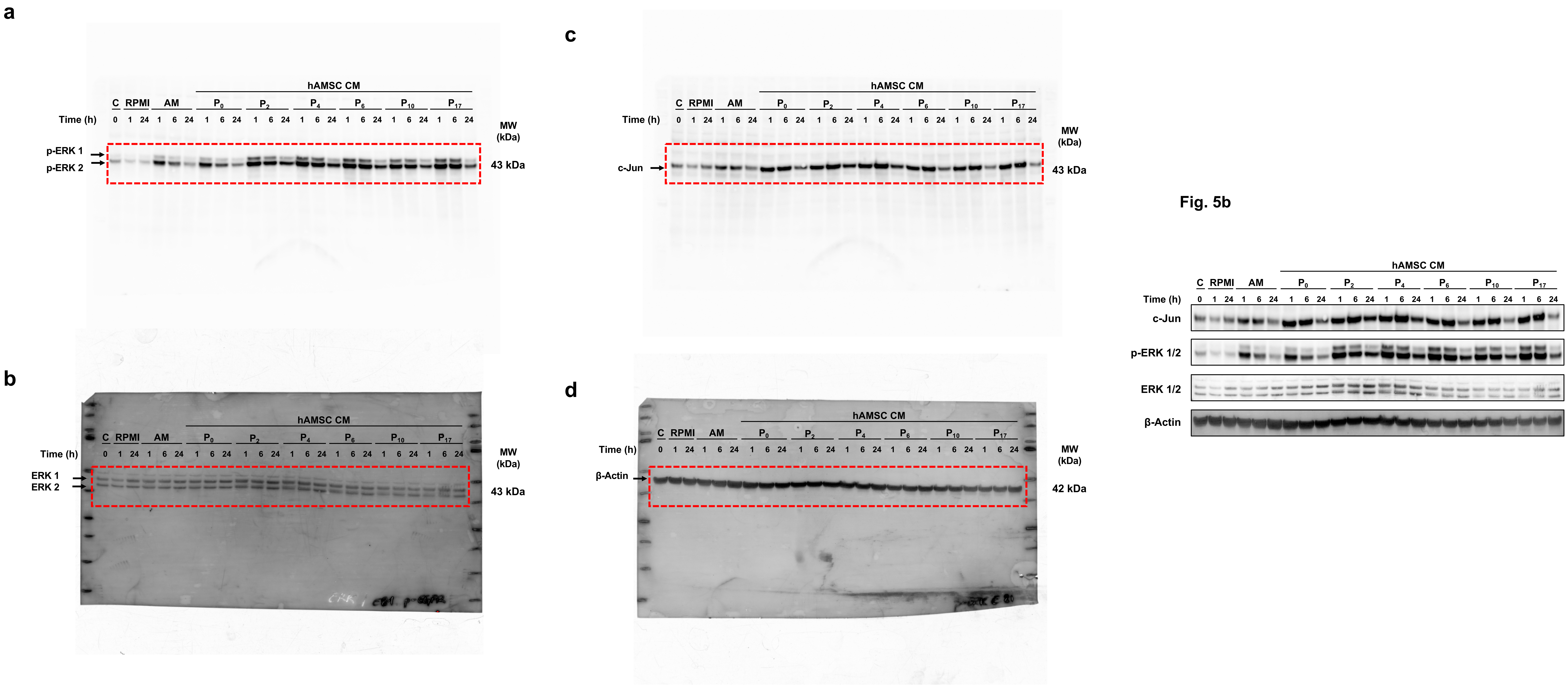

Supplemental Figure 4. Full-length blots corresponding to crops showed in Figure 5b: (a) Thr 202/Tyr 204 Phosphorylated ERK (b) ERK1/2 (c) c-Jun (d) Beta-actin loading. Dashed red rectangle indicates the portion of the blot that was used in the figure.

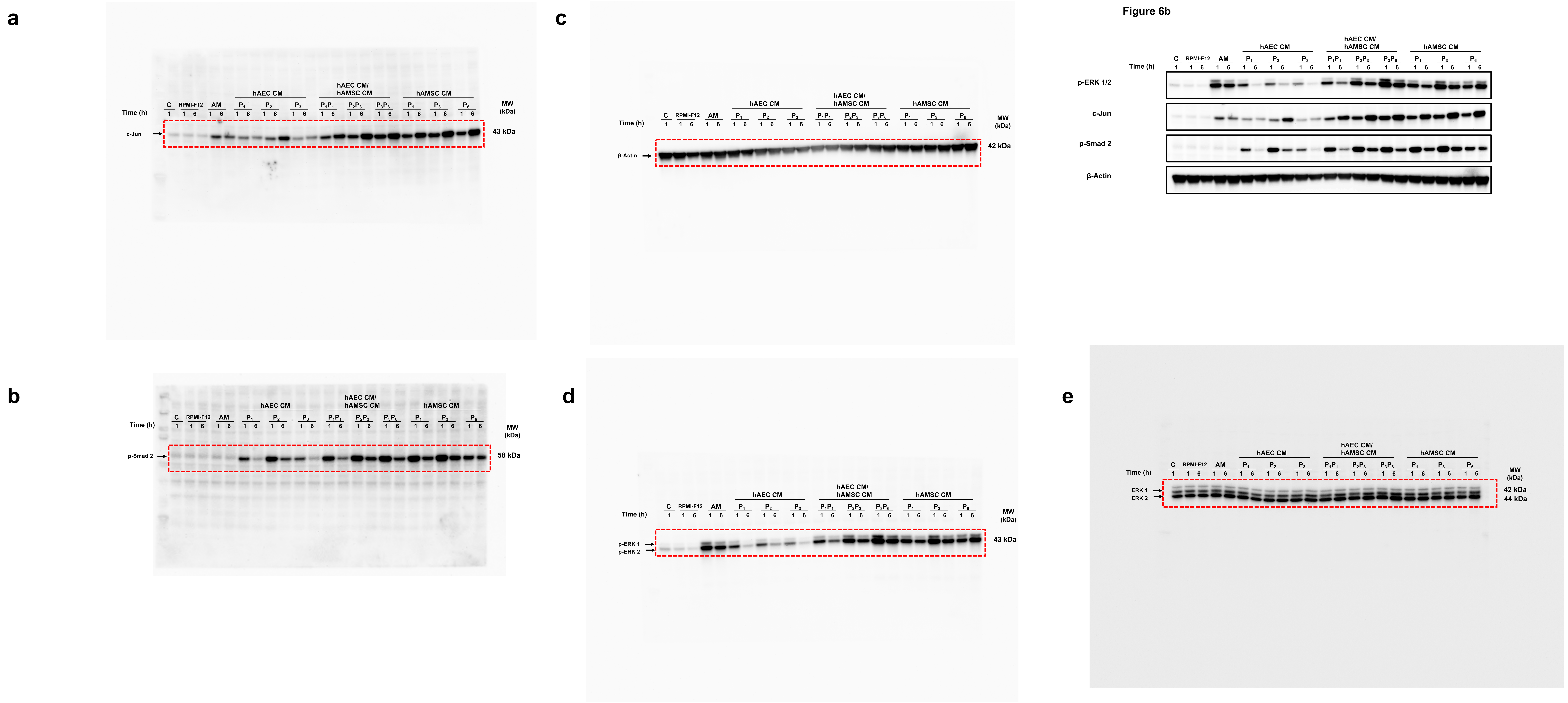

Supplemental Figure 5. Full-length blots corresponding to crops showed Figure 6b: (a) c-Jun (b) Ser465/467 Phosphorylated Smad 2 (c) Beta-actin loading (d) Phospho-p44/42 MAPK (Erk1/2)(Thr 202/Tyr 204) (e) p44/42 MAPK (Erk1/2). Dashed red rectangle indicates the portion of the blot that was used in the figure.
