# Supplementary material for: Distinct roles of amniotic membrane epithelial (hAEC) and mesenchymal stromal cells (hAMSC) in amniotic membrane-driven wound healing
Source: Sci Rep. 2025 Oct 21;15:36806. doi: 10.1038/s41598-025-20685-4 (PMC12540749; doi:10.1038/s41598-025-20685-4)
Supplement: Supplementary file 2 — Supplementary Material 2 [file 41598_2025_20685_MOESM2_ESM.pdf]

Supplemental figure legends

Supplemental Figure 1. Conditioned media from hAEC and hAMSC stimulates phosphorylation of both ERK and c-Jun. Quantification of results. Western blots from Figure 5a and 5b were quantified, and the results are presented as bar graphs representing the intensity values of each protein assayed. Intensity values were quantified using ImageJ software and compiled. Phospho-ERK1/2 values are normalized to total ERK, while c-Jun values are normalized to β-actin. (a) Quantification of western blots from Figure 5a. (b) Quantification of western blots from Figure 5b. Asterisks denote statistically significant differences between the selected conditions, as determined by one-way ANOVA followed by Tukey's multiple comparisons test (ns: $p>0.05$, *$p<0.05$, **$p<0.01$, ***$p<0.001$, ****$p<0.0001$). N≥3.

Supplemental Figure 2. Mixes of the conditioned media from hAEC and hAMSC stimulates the migration of serum starved HaCaT. Confluent serum-starved HaCaT cells (SS-HaCaT) were scratched and treated with either hAM, hAEC-CM, hAMSC CM or mix of CM for 24 hours. F12-DMEM-RMPI is used as a control and refers to the media used for conditioning. Representative images of the wound area before treatment (0 h) and after 24 hours of treatment. Scale bar: 200 µm. N≥3.

Supplemental Figure 3. Mixes of conditioned media from hAEC and hAMSC stimulates phosphorylation of both ERK and c-Jun. Quantification of results. Bar graphs display the quantified intensity values of each protein assayed by Western blot. Intensity values were measured using ImageJ software and normalized to β-actin.

Phospho-ERK1/2, c-Jun, and phospho-Smad2 values are represented. Asterisks denote statistically significant differences between selected conditions, determined by one-way ANOVA followed by Tukey's multiple comparisons test (ns: $p>0.05$, *$p<0.05$, **$p<0.01$, ***$p<0.001$, ****$p<0.0001$). N≥3.

Supplemental Figure 4. hAMSC conditioned media promotes migration through dynamization of cytoskeletal F-actin and FAs. Confluent SS-HaCaT cells were scratched and allowed to migrate for 24 hours. Cells were treated with either hAEC-CM, hAMSC-CM, hAM or a mixture of both media before conditioning (RPMI+F12). Then, cells were immunostained with specific antibodies against paxillin (green), and co-stained for phalloidin and Hoechst-33258 that were used to reveal actin cytoskeleton (red) and nuclei (blue). Representative images are shown. Scale bar indicates 50 μm. N≥3.
